# The role of phosphorus in catalytic processes of hydrogen energy technology: a perspective

**DOI:** 10.1039/d5ra10025a

**Published:** 2026-05-13

**Authors:** Gershon Amenuvor, Juliana Mana Edor, Phillimon Modisha, Dmitri Bessarabov, Banothile C. E. Makhubela

**Affiliations:** a Department of Chemistry, Faculty of Physical and Computational Sciences, College of Science, Kwame Nkrumah University of Science and Technology, PMB, University Post Office, KNUST Kumasi Ghana gershon.amenuvor@knust.edu.gh gamenuvor@yahoo.com gershonvi@gmail.com; b HySA Infrastructure Centre of Competence, Faculty of Engineering, North-West University Private Bag X6001 Potchefstroom 2531 South Africa; c Department of Chemical Sciences, University of Johannesburg PO Box 524, Auckland Park 2006 South Africa

## Abstract

The pursuit of hydrogen energy presents a promising path toward meeting growing energy needs sustainably while addressing urgent climate issues. However, developing a hydrogen economy demands significant investments in advanced infrastructure for production, storage, and transportation. The use of critical minerals is essential at nearly every stage of hydrogen technology to ensure efficiency. Consequently, one of the key future challenges will be managing these minerals responsibly to prevent depletion. Phosphorus, for instance, plays a crucial role in research on liquid organic hydrogen storage systems and is becoming increasingly important in catalyst development for water splitting. As research in this field expands rapidly, the demand for phosphorus in hydrogen technology will inevitably rise. This review highlights phosphorus' significance in advancing hydrogen technology, covering its applications in heterogeneous photocatalysis, including black phosphorus, red phosphorus, transition metal phosphides, and emerging high-entropy phosphide materials, as well as phosphorus-doped supports for ammonia borane hydrolysis. In homogeneous catalysis, the review examines the role of phosphorus-based ligands in designing catalysts for liquid organic hydrogen carrier (LOHC) systems, particularly those involving carbon dioxide conversion into formic acid, formate, amides, and methanol. The review also addresses catalyst deactivation mechanisms, theoretical descriptors for rational catalyst design, and sustainable phosphorus management strategies including immobilization, durability, recovery, and efficiency metrics. By emphasizing phosphorus' vital contributions, this article aims to raise awareness of its role in the hydrogen economy, encourage its thoughtful integration into future technologies, and promote sustainable practices in its use.

## Abstract 2

The graphical abstract highlights phosphorus' growing role in hydrogen technology: from electrocatalysts for water splitting to phosphorus-based catalysts for hydrogen storage in LOHCs and SHCs.

## Graphical abstract

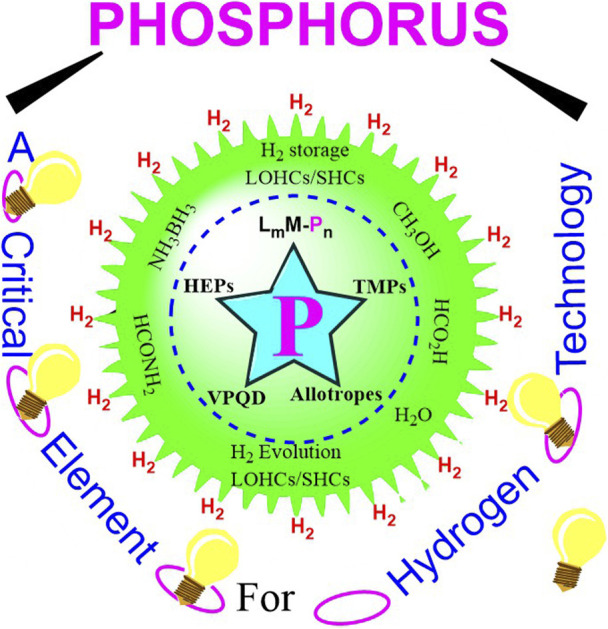

## 1. Introduction

The growing global demand for hydrogen energy has highlighted the need to utilize abundant and rare earth elements in developing efficient production technologies. As the world transitions to cleaner energy like hydrogen, the reliance on these critical materials will intensify, as they play a crucial role in every aspect of the hydrogen technology, including electrolysis, separation, storage, transportation, and fuel cell design.^1–5^ Hence, their continued use is currently unavoidable. Among these scarce but essential materials are the platinum group metals, such as iridium, platinum, palladium, and ruthenium.^4^ They are used in electrocatalysis for hydrogen generation, hydrogen storage and fuel cell make-ups. One of the most important elements that has numerous applications and in recent times, is emerging in the hydrogen economy is phosphorus. Phosphorus is among the finite elements on earth, whose depletion is raising sustainable concerns,^6^ yet its versatility is numerous.^7–9^ Currently, significant research towards hydrogen production and storage has given much attention to its use as a component of catalyst design. This review explores the role of phosphorus in hydrogen technology, particularly its use in designing phosphorus-ligated metal complexes for hydrogen evolution and storage, as well as heterogeneous catalysts for hydrogen production through water splitting. While some studies on phosphorus-based materials for hydrogen generation are not included here, readers can find a significant number of them in the referenced literature.^10^ This review does not aim to be exhaustive but rather to provide a representative cross-section of the literature, illustrating the trends and frequency with which phosphorus-based systems are being explored in hydrogen energy catalysis. Both high-performing and more modest catalysts are included to offer readers a realistic landscape of the field, highlighting that phosphorus incorporation does not guarantee superior performance and that careful ligand design remains essential.

## 2. Phosphorus-mediated heterogeneously catalyzed hydrogen evolution

Recently, phosphorus has gained considerable interest in the development of heterogeneous photocatalysts for water splitting, owing to its ability to enhance catalytic performance while maintaining thermal and corrosion stability.^11,12^

### 2.1 Black phosphorus-based heterogeneously catalysed HER

**Fig. 1:**
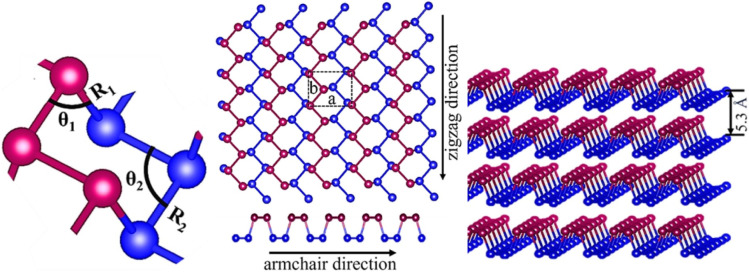
Structure of black phosphorus. (a) Structural arrangement of the atoms (b) monolayer BP (phosphorene) (c) Multilayer BP.^13^*Reproduced from ref. 13, under the terms of the Creative Commons Attribution 4.0 License (CC BY 4.0)*. *Copyright 2022, V. Chaudhary, P Neugebauer, O Mounkachi, S Lahbabi, and A El Fatimy.*

Among its allotropes, black phosphorus (BP), the most stable form, has emerged as a key material in photocatalyst design. As a two-dimensional layered semiconductor (Fig. 1),^13^ BP offers tunable band gaps (0.3 eV in bulk to 2.0 eV in monolayer phosphorene), high charge carrier mobility, and other advantageous properties, including pronounced crystal anisotropy and a balanced on/off ratio.^14–16^ Given these modifiable features, it is no surprise that BP is being actively explored as a promising catalyst for hydrogen evolution, with potential for high efficiency. BP can be synthesized from its allotropes (white or red phosphorus) using high-pressure and high-temperature methods,^17^ chemical techniques like chemical vapor transport and solvothermal reactions,^18–21^ or alternative approaches such as mechanical and liquid exfoliation.^22–24^

**Fig. 2:**
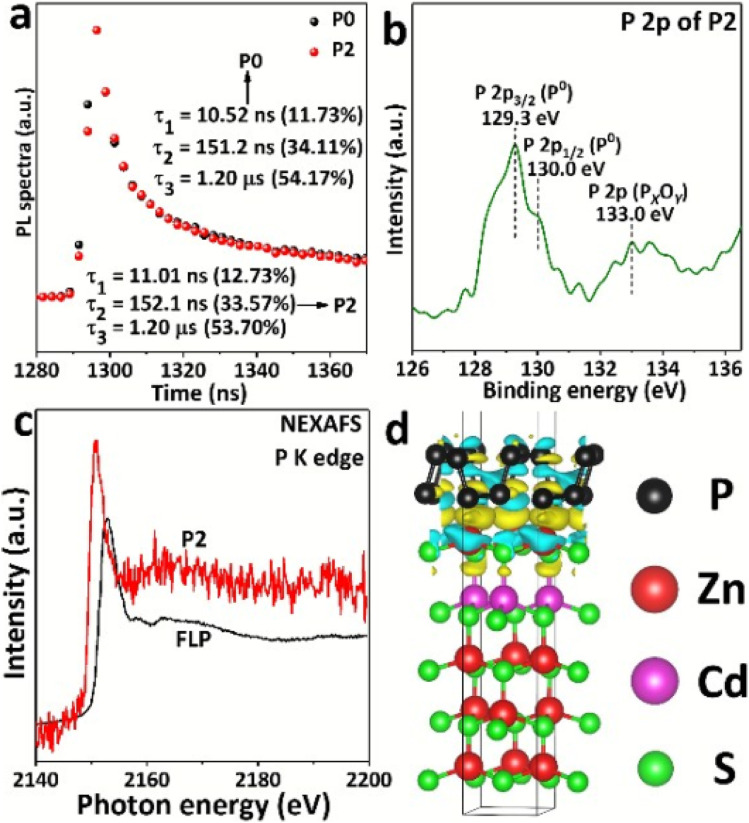
Characterization of the materials; images used with permission (a) transient-state photoluminescence spectra from P0 to P2. (b) High resolution XPS spectrum of P 2p for P2; (c) NEXAFS P K edge of FLP and P2; (d) side view differential charge density map of ZCS/FLP material.^25^*Reproduced from ref. 25 with permission from the Royal Society of Chemistry, Chem. Commun., 2017,****53****, 9882, Copyright 2017*.

Black phosphorus can enhance the photocatalytic performance of other semiconductors, improving their overall efficiency. Researchers have taken advantage of the suitable forbidden bandwidths possessed by metal sulfides to design hybrid BP-metal sulfide photocatalysts. In comparison to the bare Zn_*x*_Cd_1−*x*_S (ZCS) nanoparticles, Qiao *et al.*^25^ demonstrated that few-layer phosphorene nanosheets (FLP) decorated on the metal sulfides ZCS are superior photocatalysts for hydrogen production from water. When 0.5 wt% FLP nanosheets were deposited on ZCS nanoparticles, the hydrogen production under visible light increased to 1476 µmol h^−1^ g^−1^, which is significantly higher than the 484 µmol h^−1^ g^−1^ achieved by pure ZCS. The highest activity of 9326 µmol h^−1^ g^−1^, was obtained with a 2.0 wt% FLP nanosheets loading. Analysis of the material revealed that electron transfer occurred from ZCS to the FLP, enhancing the activity of the ZCS/FLP system (Fig. 2). In a related study, Yuan and coworkers^26^ fabricated a 2D/2D BP/MoS_2_ heterojunction composite, which exhibited a high hydrogen evolution rate of 1286 µmol h^−1^ g^−1^ under visible light (*λ* > 420 nm) when the composite constituted 10% of the photocatalyst. When irradiation energy was slightly reduced (*λ* > 550 nm), catalytic performance dropped significantly, yielding only 341 µmol h^−1^ g^−1^ of hydrogen. Majima and coworkers also reported the application of BP/WS_2_ hybrid photocatalyst with NIR laser light irradiation (>780 nm) for hydrogen evolution from water, and observed 50-fold catalytic activity (2.3 µmol h^−1^ g^−1^) over the individual BP and WS_2_ materials.^27^

Research has shown that combining BP with metal oxides significantly improves both its recyclability and photocatalytic efficiency. A typical successful example is the 2D/2D BP/Bi_2_WO_6_ heterostructure (BP/MBWO) developed by Chen's research team, which demonstrated enhanced hydrogen production *via* water splitting.^28^ The photocatalytic performance of the first cycle using 12 wt% BP/MBWO in the presence of H_2_PtCl_6_·6H_2_O as a co-catalyst under visible light irradiation reached 21 042 µmol g^−1^. However, the pristine MBWO exhibited significantly inferior performance, achieving only about one-ninth of the hybrid material's efficiency.

Several other metal-incorporated BP hybrid materials have demonstrated photocatalytic hydrogen evolution through water splitting.^29,30^ Notably, Yu *et al.*^29^ developed a TiF_3_-BP composite system consisting of polycrystalline BP nanosheets enhanced with a titanium fluoride cocatalyst. This catalyst performed well in water splitting under ultraviolet-visible light irradiation, recording a high activity of 612 µmol h^−1^ g^−1^. Compared to pure BP and pure TiO_2_, the evolution rate of hydrogen by the TiF_3_-BP hybrid composite was almost 2 times higher. Furthermore, the hybrid material's performance surpasses that of commercial P25-BP mixtures by approximately 1.5 times.

Studies demonstrate that the photocatalytic efficiency of BP is strongly dependent on its structural properties. Yang *et al.*^30^ observed that untreated bulk BP showed limited visible-light photocatalytic activity for water splitting, achieving only 20 µmol h^−1^ g^−1^ hydrogen evolution. However, when processed through mechanical ball-milling with LiOH to produce BP-BM nanosheets, the activity increased dramatically to 512 µmol h^−1^ g^−1^. Interestingly, adding 3 wt% Pt (Pt/BP-BM) under identical conditions reduced the hydrogen production to 224 µmol h^−1^ g^−1^.

### 2.2 Elemental red phosphorus catalyzed HER

**Fig. 3:**
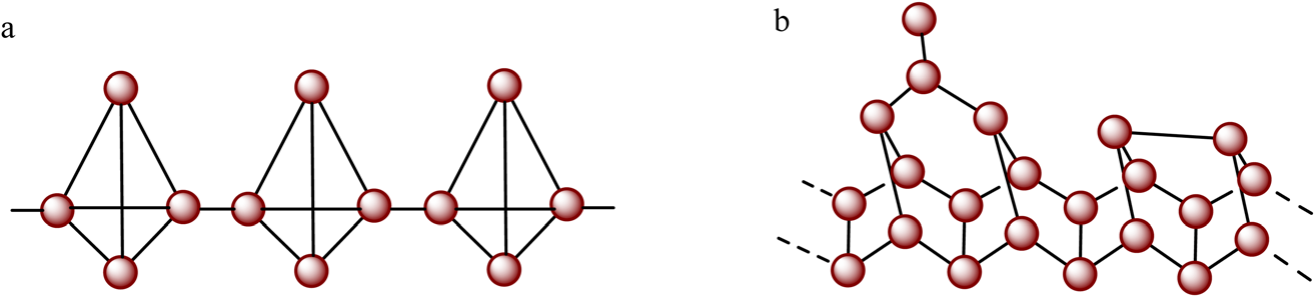
Structure of (a) red phosphorus and (b) violet phosphorus.

Elemental red phosphorus (RP) has also attracted considerable attention as a metal-free photocatalyst for hydrogen production due to its natural abundance, broad light absorption capability, and favorable band structure for photocatalytic reactions.^31,32^ Early progress in this field was reported in 2012 when Wang and co-workers^32^ demonstrated the first example of photocatalytic hydrogen generation using crystalline RP prepared by heating amorphous RP at 450 °C for 12 h. They achieved a hydrogen evolution rate of 0.08 µmol h^−1^ and further demonstrated that the introduction of 1 wt% Pt cocatalyst enhanced the H_2_ evolution rate over 10 times, highlighting the importance of cocatalyst-assisted charge separation in RP systems.^32^ Since then, significant efforts have been devoted to improving the photocatalytic efficiency of RP-based materials for the HER. Fig. 3 illustrates the structures of red and violet phosphorus allotropes.

While white and red phosphorus typically exhibit poor thermal stability compared to BP, Yu's team made a breakthrough discovery,^33^ demonstrating exceptional catalytic activity from processed fibrous RP. Their optimized RP/SiO_2_ composite (with uniform distribution on photo-inactive SiO_2_) achieved a remarkable 684 µmol h^−1^ g^−1^. In general, these RP-based systems significantly outperform conventional metal-free photocatalysts like Pt-modified g-C_3_N_4_ (107 µmol h^−1^ g^−1^),^34^ highlighting RP's superior potential for hydrogen evolution reactions. Further improvements have been realized through the construction of heterostructured systems. For example, Dai and coworkers^35^ developed an RP/TiO_2_ composite using the chemical vapor deposition strategy. The optimized RP/TiO_2_ heterostructure exhibited a hydrogen evolution rate of 681 µmol h^−1^ g^−1^. This enhanced performance was attributed to the formation of interfacial Ti–O–P bonds at the heterojunction, which promoted strong interfacial coupling between TiO_2_ and RP. Such bonding facilitates efficient charge separation while suppressing electron–hole recombination and self-trapping within the TiO_2_ component.^35^ More recently, in 2023, Wang *et al.*^36^ reported a novel photocatalytic system based on violet phosphorus (VP), an allotrope derived from RP. By synthesizing VP quantum dots, the authors achieved exceptionally high photocatalytic activity, with a hydrogen evolution rate of 3325.1 mmol h^−1^ g^−1^, representing one of the highest performances reported for mono-elemental photocatalysts. These advances highlight the rapidly growing potential of phosphorus-based materials as efficient metal-free photocatalysts for solar-driven hydrogen production (Table 1).

**Table 1:** Summary of selected elemental phosphorus-based heterogeneous photocatalysts for hydrogen evolution

Catalyst composition	Irradiation (nm)/QY^a^ (%)	H_2_ evolution rate (µmol h^−1^ g^−1^)	Ref.
FLP/ZCS	420/21.5	9326	25
BP-1000/MoS_2_	420/1.2	1286	26
BP/WS_2_	>780/2.06	2.3	27
BP/Bi_2_WO_6_	—	21 042	28
TiF_3_-BP	UV-vis	612	29
BP-BM	420/0.47	512	30
RP-1wt% Pt	400	0.950	32
RP/SiO_2_	>420	684	34
RP/TiO_2_	≥420 nm	681	35
VPQD	≥420 nm	3325.1	36

a QY = quantum yield.

### 2.3 Transition metal phosphides photocatalytic hydrogen production

In photocatalytic hydrogen production, cocatalysts play a crucial role in enhancing both the efficiency and durability of the photocatalysts. They contribute by enhancing light absorption, facilitating charge separation and transfer, increasing the overall stability of the photocatalytic system, and delivering active sites for the reaction.^37,38^ Transition metal phosphides (TMPs) composed of earth-abundant elements have also emerged as cost-effective and highly efficient catalysts for the HER, attracting significant research interest. In 2005, Liu and Rodriguez^39^ predicted, through density functional theory (DFT) calculations, that Ni_2_P could serve as a highly active HER catalyst. This was later experimentally validated in 2014 when Cao *et al.*^40^ demonstrated that Ni_2_P functions as an excellent cocatalyst for photocatalytic hydrogen production. Since then, considerable research efforts have focused on incorporating TMPs into photocatalytic systems for H_2_ generation.^41–45^ The electronic structure of TMPs plays a critical role in their catalytic activity for HER.^39,46^ Phosphorus possesses a relatively high electronegativity compared with most transition metals, which induces a partial negative charge on the P-atoms within the metal–phosphide lattice. These negatively polarized P sites can electrostatically attract protons (H^+^) from the reaction medium, facilitating proton adsorption during the HER process. Meanwhile, the adjacent metal atoms act as electron donors that promote the reduction of the adsorbed protons to hydrogen.^47^ This cooperative interaction between proton-accepting P sites and electron-rich metal centres creates an efficient pathway for hydrogen evolution. In addition to enhancing catalytic activity, the strong covalent character of the metal–phosphorus bonds contributes significantly to catalyst stability.^48^ These covalent interactions stabilize the crystal lattice and prevent metal aggregation or dissolution under reaction conditions, thereby improving the durability of TMP catalysts in photocatalytic and electrocatalytic systems.^41^ As a result, TMPs such as Ni_2_P, CoP, and FeP exhibit both high catalytic activity and excellent structural robustness for hydrogen production.

### 2.4 High-entropy phosphide catalysts

An emerging frontier in phosphorus-based heterogeneous catalysis is the development of high-entropy phosphide (HEP) materials, which combine five or more metal elements in a single phosphide phase to achieve synergistic catalytic effects beyond those of binary or ternary phosphides.^49^ This approach leverages the high-entropy stabilization effect to create catalysts with unique electronic structures and enhanced stability.^49^

Using a nickel foam-supported high-entropy phosphide (FeCoNiCuMnP/NF), Zhao and colleagues achieved an alkaline methanol oxidation system that reaches a current density of 10 mA cm^−2^ at just 1.32 V while maintaining exceptional selectivity toward formate products.^50^ The origin of this enhanced performance was traced through Monte Carlo simulations to the unique phosphorus coordination environment within the high-entropy lattice, where the varied metal-phosphorus bonding arrangements give rise to electronic states optimally tuned for substrate adsorption and activation.^50^

The rational design of HEP catalysts builds upon extensive research in transition metal phosphides (TMPs), which have been established as promising non-precious metal electrocatalysts for hydrogen evolution reactions (HER).^51^ Metal doping has become a commonly used method for modifying TMPs because it enables precise control over doping levels and offers simple synthesis routes.^51^ Metal atom doping fundamentally alters the intrinsic properties of TMPs, including the electrochemically active surface area and electronic structure, while the construction of composite materials such as NiCoP/rGO can further enhance catalytic activity.^51^

Polymetallic doping represents a natural progression toward high-entropy systems. Examples such as CoMo(Al)-P demonstrate that synergy between multiple metals can greatly improve catalytic performance.^52^ The extension of this concept to high-entropy alloys and phosphides opens new avenues for catalyst development, with early results suggesting that HEPs may offer superior activity and stability compared to their lower-entropy counterparts.^50,52^

Notably, TMPs and their high-entropy derivatives also show promise for seawater electrolysis applications. For instance, Fe-Co_2_P branched nanorods have demonstrated good catalytic activity in seawater, expanding the potential operating environments for hydrogen production.^53^

The tunable composition of HEPs allows systematic optimization of catalytic properties for hydrogen evolution and oxidation reactions, representing a promising direction for developing cost-effective, noble-metal-free catalysts.^50,52^ This emerging field illustrates how phosphorus continues to enable new catalytic paradigms beyond traditional binary phosphides and single-metal phosphide systems.

### 2.5 Phosphorus-doped heterogeneous catalysts for ammonia borane hydrolysis

In recent years, phosphorus-doped heterogeneous catalysts have attracted increasing attention for ammonia borane (AB) hydrolysis due to their ability to modulate the electronic structure of catalytically active sites, stabilize, and enhance metal-support interactions.^54,55^ Incorporation of phosphorus into catalyst supports such as carbon materials, metal carbides, or metal oxides introduces electron-rich P species that can improve metal dispersion, modify the adsorption properties of AB and water molecules, and facilitate the cleavage of B–H and O–H bonds during the hydrolysis.^55^ One representative example is the use of rhodium nanoparticles supported on phosphorus-doped porous carbon (Rh/PPC), which exhibited excellent catalytic performance for AB hydrolysis with a TOF of 806 min^−1^. Notably, the presence of P-containing surface functionalities enhanced the interaction between Rh precursors and the support, leading to the formation of ultrasmall, well-dispersed metal nanoparticles and improved access to active sites.^55^ Wan *et al.*^56^ developed a photocatalytic system for AB hydrolysis by utilizing Ni–Pt nanoparticles supported on phosphorus-doped TiO_2_ (Ni-Pt/P-TiO_2_). Impressively, the optimized catalyst (Ni_40_Pt_60_/P-TiO_2_) resulted in a high TON of 967.8 mol_H2_ mol_Pt_^−1^ min^−1^, attributed to the synergistic properties of the Ni and Pt sites as well as the strong metal–support interactions. Moreover, comparing the performance of Ni_40_Pt_60_/TiO_2_ and Ni_40_Pt_60_/P-TiO_2_ under identical conditions revealed that phosphorus doping enhances visible-light absorption, promotes efficient photoinduced electron transfer, and alters the electronic structure of the metal centers, thereby strengthening metal–support interactions and significantly improving both catalytic activity and stability.^56^

Furthermore, phosphorus-doped transition-metal carbide systems have been explored as efficient noble-metal-free catalysts. For example, Ni nanoparticles supported on a P-doped Mo@Mo_2_C heterostructure (Ni/P-Mo@Mo_2_C) resulted in high activity for AB hydrolysis with a TOF of 222 min^−1^ at 298 K, which increased to 413 min^−1^ under alkaline conditions. The enhanced activity was attributed to the electronic modulation induced by P doping and the strong metal–support interaction that promotes the dissociation of water molecules in the rate-determining step of the hydrolysis reaction.^57^ In addition to carbon and carbide supports, phosphorus-containing oxide materials have also been investigated as active catalyst supports. For instance, Pt nanoparticles supported on hydroxyapatite (Ca_10_(PO_4_)_6_(OH)_2_) have demonstrated efficient catalytic hydrolysis of AB. In this system, the coexistence of Lewis acidic Ca^2+^ sites and Lewis basic phosphate groups facilitates substrate activation and enhances hydrogen evolution performance.^58^

Overall, these studies demonstrate that phosphorus incorporation into heterogeneous catalysts is an effective strategy for enhancing catalytic performance, primarily by tuning the electronic structure of active sites, stabilizing metal nanoparticles, and promoting key reaction steps such as water activation and hydrogen evolution. Continued advances in phosphorus-doped materials are therefore expected to play an important role in the development of efficient and cost-effective catalysts for chemical hydrogen storage systems.

## 3. Phosphorus-mediated homogeneously catalyzed hydrogen storage and release

Phosphorus-based molecular complexes have emerged as effective homogeneous catalysts for hydrogen storage and release applications. These systems predominantly facilitate catalytic transformations of liquid organic hydrogen carriers (LOHCs), including formic acid, methanol, and various cyclic organic compounds. Notably, some of this class of catalysts demonstrate dual functionality, capable of both hydrogenating and dehydrogenating LOHCs, a crucial feature for developing sustainable energy storage systems.^59^

This discussion highlights significant advances in hydrogen energy technology employing phosphorus ligands as the central components controlling the catalytic activity of LOHC systems, specifically those comprising CO_2_-formic acid/methanol/amide switch.

### 3.1 CO_2_ hydrogenation and formic acid dehydrogenation catalysis

**Scheme 1:**
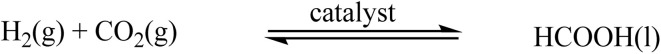
Reversible catalytic CO_2_ hydrogenation.

Recent decades have seen extensive studies on phosphorus-based metal complexes catalyzing formic acid dehydrogenation and CO_2_ hydrogenation (Scheme 1).

An early breakthrough was achieved by Leitner and Graf, who demonstrated the effective catalytic hydrogenation of CO_2_ to formic acid at ambient temperature using a system comprising [{Rh(COD)CI}_2_] and a bidentate phosphine ligand, DPPB (DPPB = Ph_2_P(CH_2_)_4_PPh).^60^ At optimal reaction conditions of 40 atm total pressure (H_2_ : CO_2_ = 1 : 1), 1.35 × 10^−3^ M rhodium concentration, 72 µmol DPPB, 7.21 mmol triethylamine and 22 h, the group obtained 1150 mol of formic acid per mole of rhodium used (*i.e.*, Turnover number, TON of 1150). Control experiments revealed that formic acid production ceased entirely without DPPB, with metallic rhodium deposition observed instead. This provides direct evidence that the bidentate phosphine ligand (Ph_2_P(CH_2_)_4_PPh_2_) is essential for both stabilizing the rhodium catalyst in solution and preventing its decomposition to inactive metallic species. Under ambient pressure, the reverse reaction to release hydrogen and carbon dioxide was observed at a comparable rate to that of the forward reaction. Using water-soluble triphenylphosphine trisulfonate (TPPTS) with the rhodium precursor [{Rh(COD)CI}_2_], Leitner's group recorded an improved TON of 3439, which is almost three times higher than what was achieved with DPPB.^61^ The *in situ* formed rhodium-phosphine catalyst systems developed by Leitner's group provided critical insights into how phosphine ligands facilitate the reversible catalytic cycle between carbon dioxide and formic acid, highlighting their indispensable role in these transformations. Although earlier studies had explored phosphine-coordinated complexes like Wilkinson's catalyst (RhCl(PPh_3_)_3_),^62,63^ research in this domain remained limited prior to this work. In 2017, Leitner's group again revealed a highly efficient catalytic system that converted CO_2_ through hydrogenation to formate-amine adducts in a biphasic system using *cis*-[Ru(DPPM)_2_Cl_2_] (DPPM = bis-diphenylphosphinomethane) (1, Fig. 2) in the presence of triethylamine.^64^ The catalytic system exhibited exceptional activity and robust stability in both hydrophobic ionic liquid media (1-methyl-3-octylimidazolium bis(trifluoromethylsulfonyl)imide) and organic phases such as methyl isobutyl carbinol (MIBC). The MIBC/water system demonstrated superior catalytic performance, achieving an exceptional initial turnover frequency (TOF) of 180 000 h^−1^ and maintaining a high average TOF of 35 000 h^−1^ throughout the reaction. Furthermore, this optimized system exhibited minimal ruthenium leaching, with less than 0.26% loss per catalytic cycle. Using the MIBC system, a total turnover number (TTON) of 150 000 was achieved over 11 cycles when monoethanolamine was used as a base, while Aminosol CST 115 yielded a TTON of 18 170 over 10 cycles.

In a 2018 review, Sordakis *et al.*^65^ comprehensively examined homogeneous catalysts for hydrogen storage in formic acid and alcohols. Notably, their data revealed that phosphorus-containing ligands dominated the field, accounting for approximately 90% of reported catalysts for CO_2_ hydrogenation and hydrogen release. These phosphorus-based systems encompassed both monophosphine- and bisphosphine-multidentate mix-donor ligands, employed either as pre-synthesized complexes or formed *in situ* during catalysis. Following this review, subsequent research has continued to explore and develop analogous phosphorus-ligated catalytic systems.^67–77^

**Fig. 4:**
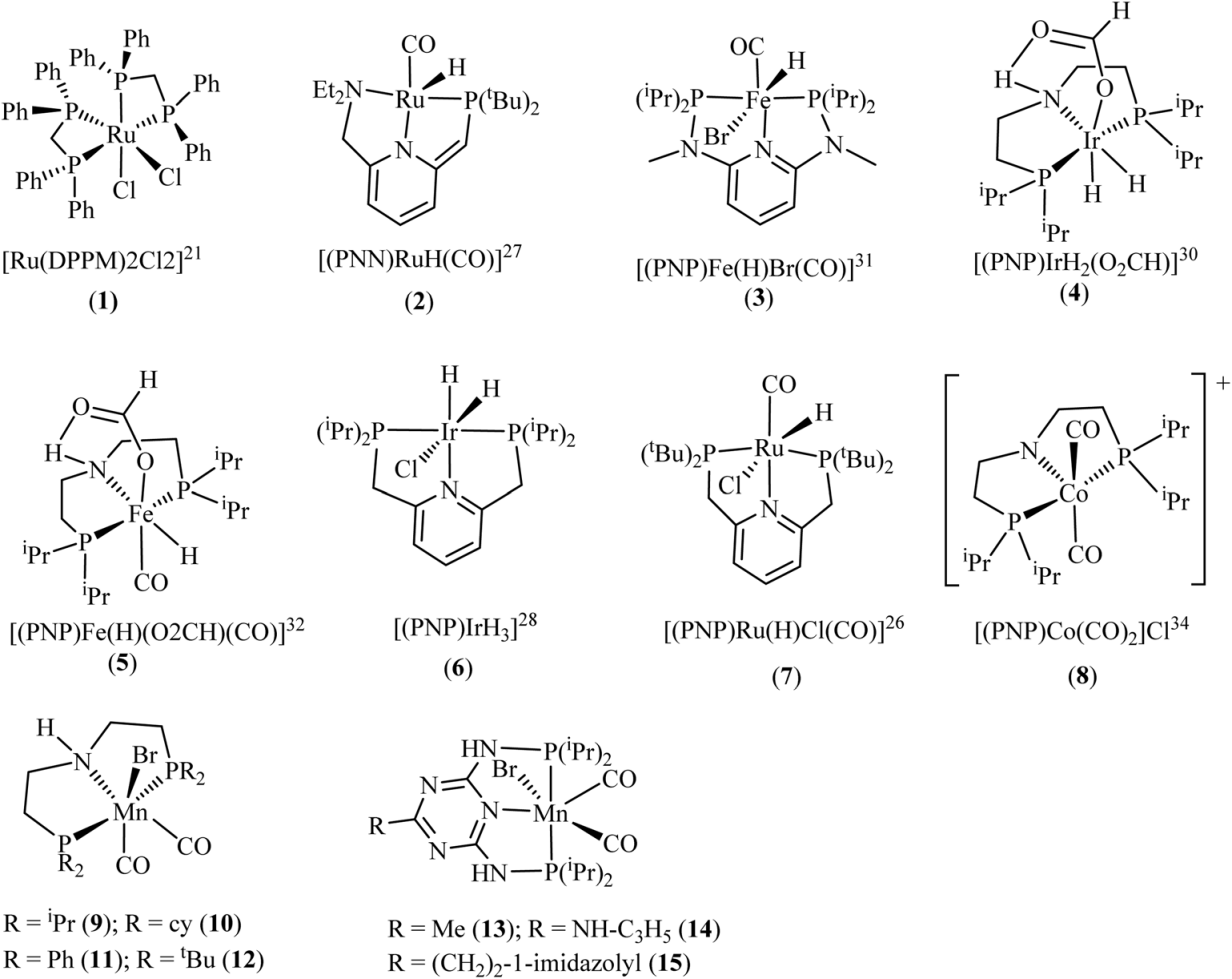
Examples of very active complexes bearing phosphine ligands for catalytic CO_2_ hydrogenation to formic acid. Please refer to Table 2 for their performance.

Some of such catalysts that showed impressive catalytic activity for CO_2_ conversion to formic acid are shown in Fig. 4 and Table 2.

**Table 2:** Examples of phosphorus-based ligand/complexes previously used for CO_2_ hydrogenation with their performance. This table is a modified version (including new data) of previously reported work.^65^ NB: Not all TOFs were obtained from the TONs in the table

Catalyst precursor	Solvent	Base	CO_2_/H_2_ (bar)	*T*/°C	Time/h	TON	TOF
[*cis*-Ru(DPPM)_2_Cl_2_]^37^	MIBC/H_2_O	Aminosol CST 115	30/60	70	3 min	150 000	180 000
[RuH_2_(PMe_3_)_4_]^40^	scCO_2_	NEt_3_/DMSO (or MeOH)	120/85	50	0.5	2000	4000
[RuCl(OAc)(PMe_3_)_4_]^41^	scCO_2_	NEt_3_/C_6_F_5_OH	120/70	50	0.3	31 200	95 000
[RuCl_2_(*m*TPPMS)_2_]_2_/*m*TPPMS^42^ (7)	H_2_O	NaHCO_3_	35/60	80	1	9600	9600
[(PNP)Ru(H)Cl(CO)]^43^ (2)	DMF	DBU	10/30	120	0.1	200 000	1 100 000
[(PNN)RuH(CO)]^44^ (6)	Diglyme	K_2_CO_3_	10/30	200	48	23 000	480
[(PNP)IrH_3_]^45^	H_2_O/THF	KOH	30/30	120	40	3 500 000	73 000
[(PNP)IrH_2_(O_2_CH)]^46^ (4)	H_2_O	KOH	28/28	185	2	37 300	18 600
[(PNP)Fe(H)Br(CO)]^47^ (3)	EtOH	DBU	40/40	80	21	10 300	500
[(PNP)Fe(H)(OOCH)(CO)]^48^ (5)	THF	DBU/LiOTf	35/35	80	1	46 100	23 200
[Co(DMPE)_2_H]^49^	THF	Verkade's base	10/10	21	2 min	9400	74 000
[(PNP)Co(CO)_2_]Cl^50^ (8)	CH_3_CN	DBU/LiOTf	35/35	45	1	29 000	5700

Phosphine-ligated Mn-pincer complexes reported by Beller and coworkers^66^ for the amino acid-promoted reversible hydrogenation of CO_2_ to formic acid were among the recently reported highly efficient systems. The catalytic performance of the Mn-pincer complexes 9–15, synthesized from diverse substituted phosphine ligands, showed significant variation in performance when combined with lysine, ranging from outstanding to ineffective, based on the specific substituents attached to the phosphorus centers. For instance, at standard reaction conditions of 80 bar (CO_2_/H_2_ = 1 : 3), 0.1 µmol catalyst loading, 5.0 mmol lysine, 10 mL water/tetrahydrofuran (1 : 1), and 145 °C, complexes 9–11 produced yields of 80, 76, and 50 respectively, translating to corresponding TONs of 40 000, 38 000 and 25 000 achieved in 12 h. In contrast, complex 12, featuring a *tert*-butyl group at the phosphorus center, produced no detectable formate. Under the same conditions, complexes 13, 14, and 15 performed slightly better, resulting in yields of 86, 88, and 86% respectively, with 14 achieving the highest TON of 44 000. Under optimized reaction conditions, a significantly higher TON of 230 000 was attained using only 0.02 µmol of 14, corresponding to a 92% yield. In addition, employing 0.17 µmol of 14 and 1.0 equivalent of lysine at 90 °C for 12 h, in FA dehydrogenation yielded 99% conversion, equivalent to a TON of 29 400. An effective homogeneous catalyst should remain stable under harsh conditions, such as high temperatures, and maintain efficiency over multiple cycles. These Mn-pincer catalysts demonstrated exceptional robustness, particularly complex 14, which was recycled ten times while retaining over 90% yield.

#### 3.1.1 Mechanistic considerations

**Scheme 2:**
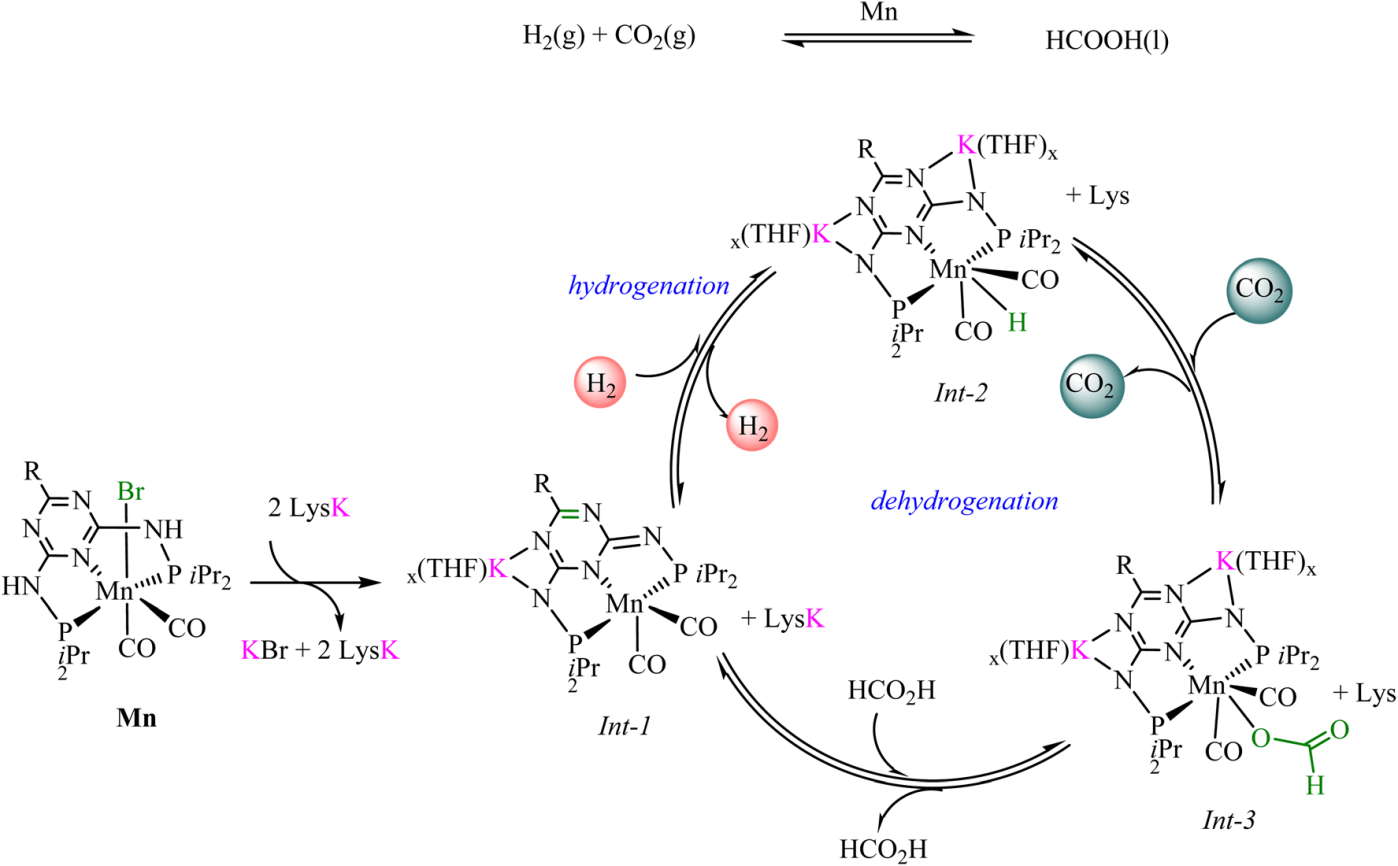
Catalytic cycle proposed for the FA/CO_2_ reversible dehydrogenation catalyzed by phosphorus-based Mn complexes.^66^*Adapted from ref. 66, under the terms of the Creative Commons Attribution 4.0 License (CC BY 4.0). Copyright 2022, D. Wei, R. Sang, P. Sponholz, H. Junge, and M. Beller.*

To probe the interplay between the catalyst and activity, Beller *et al.*^66^ monitored the key intermediates using *in situ* NMR studies, which revealed that LysK activates the Mn–PN_5_P^*i*^Pr (Mn–PN_5_P^*i*^Pr = 13–15) catalyst through the deprotonation of the N–H and triazine dearomatization, producing a Mn–K intermediate (Int-1). Exposure to H_2_ generates a Mn–hydride species (Int-2), which reacts with CO_2_ to form a Mn–formate intermediate (Int-3). Formic acid release regenerates the active species, completing the CO_2_ hydrogenation cycle. The reverse pathway enables formic acid dehydrogenation to H_2_ and CO_2_. Control studies showed that both the α-amino acid structure and a basic side chain in the amine promoter are essential for optimal hydrogenation and CO_2_ capture. Scheme 2 illustrates the proposed catalytic cycle.

### 3.2 CO_2_ hydrogen to methanol and methanol dehydrogenation

**Fig. 5:**
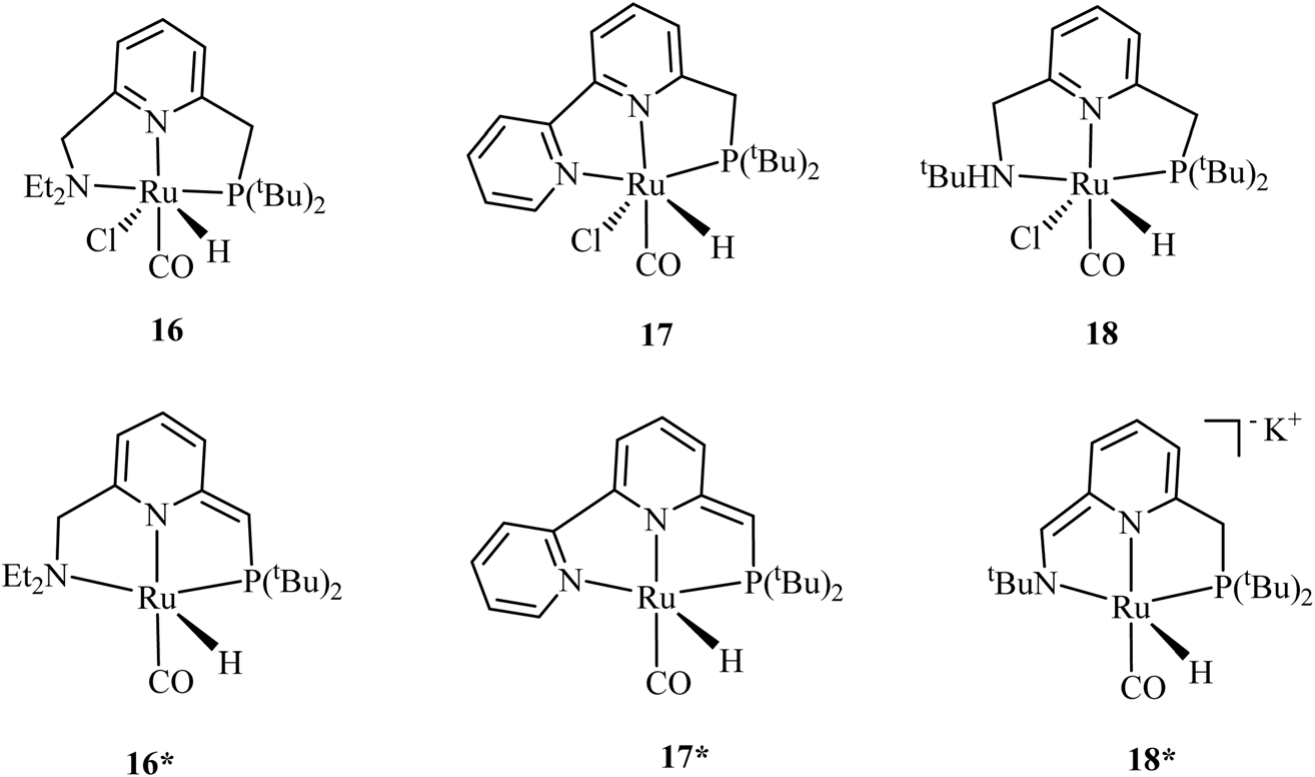
Some of the phosphorus-based catalysts used by Milstein for CO_2_ conversion to methanol.^80^*Adapted with permission from**ref*. *80**from the American Chemical Society, ACS Catal., 2015,****5****, 2416, Copyright 2015.* Prakash and coworkers^79^ recently reported a CO_2_ capture system using polyamines and subsequently converted the CO_2_ to methanol using a series of PNP-ligand-based Ru-pincer complexes 16^*^ and 19–25 (Fig. 6). The catalysts' performance varied significantly depending on the substituents at the phosphorus center. Under optimized reaction conditions (10 µmol catalyst, 5.1 mmol PEHA, 1 mmol K_3_PO_4_ in 10 mL triglyme, 75 bar CO_2_/H_2_ [3 : 1], 145 °C, 40 h), catalysts 19, 22, 23, 24, and 25 achieved turnover numbers (TONs) of 1050, 1040, 320, 50, and 680 respectively for methanol production. Surprisingly, complex 16* showed no catalytic activity under these conditions. To evaluate catalyst stability, the most effective Ru-Macho-BH (19) was tested in a continuous 10-day reaction, achieving a TON of 9900, confirming its resistance to deactivation over extended periods.^81^

The conversion of CO_2_ to methanol, an additional step following the hydrogenation of CO_2_ to formic acid or formate, is a kinetically challenging step rather than a thermodynamic issue.^78^ An effective catalyst must overcome this kinetic barrier to facilitate the transformation of formic acid into methanol. Typically, the formate intermediate binds strongly to the metal catalyst, forming a stable metal-formate complex. Thus, hydride complexes capable of readily transferring hydride to the carbonyl group are preferred for this reaction.^79^ The formate release step is usually assisted by amines to form an amide, which undergoes further reduction steps to methanol.^79^ Among the limited number of catalysts reported to perform this transformation with reasonable efficiency, those featuring phosphorus-donor ligands are the most prevalent. In 2015, Milstein's research team demonstrated an indirect CO_2_-to-methanol conversion pathway using ruthenium–pincer complexes 16–18 (Fig. 5) coupled with amino alcohols such as aminoethanol and valinol, achieving impressive yields of 78–92%.^80^ The transformation proceeded through a two-step process. First, CO_2_ was captured under ambient pressure conditions (1–3 bar) by the amino alcohols in the presence of CsCO_3_ as a catalyst, forming oxazolidinone intermediates (65–70% yield with aminoethanol, 90–95% with valinol). Subsequent hydrogenation of these intermediates at 150 °C over 24 h liberated methanol while regenerating the amino alcohol. Notably, when valinol was employed as the CO_2_-capturing agent under these conditions, the highest oxazolidinone yields were attained. Among the tested catalysts, complex 17 exhibited superior performance, generating methanol in 92% yield when the reaction was conducted in THF, though this decreased to 78% in DMSO solvent systems.

The study demonstrated how subtle structural modifications, particularly at the phosphorus centers, dramatically influenced the catalysts' effectiveness in the CO_2_ hydrogenation process. Particularly, phenyl-substituted phosphine ligands demonstrated exceptional performance, as evidenced by the high TONs obtained for complexes 19, 22, 23, and 25. In contrast, catalysts with bulky phosphine substituents (^*i*^Pr, Cy, ^*t*^Bu) exhibited reduced methanol yields. These formed biscarbonyl complexes with lower dissociation tendencies, hindering the formamide hydrogenation step.^81^ Deactivation was attributed to the formation of ruthenium biscarbonyl monohydride intermediates ([RuHPNP(CO)_2_]^+^) (26), generated from CO byproducts during the reaction. The stability of the axial CO ligand in these species was found to be a critical factor in catalyst longevity. Interestingly, catalytic activity was not solely dependent on the presence of metal hydride precursors or carbonyl ligands, as 25 performed well despite lacking both.^81^

A year later, Prakash and coworkers^82^ screened a library of tertiary amines for CO_2_ capture and its subsequent conversion to methanol using complex 19. The tertiary amines, including tetramethylethylenediamine (TMEDA) and tetramethyl-1,3-butanediamine (TMBDA) in ethylene glycol, efficiently captured CO_2_ at ambient conditions through the formation of alkyl carbonate salts. This was followed by hydrogenation of the carbonate salts to methanol using the Ru-Macho-BH catalyst (19) at 70 bar H_2_ at 140 °C, achieving yields up to 92% with TMEDA.

### 3.3 Access to methanol and hydrogen *via* amide-based CO_2_ products

Recent studies demonstrated that formamides serve as efficient liquid hydrogen carriers, capable of releasing high-purity hydrogen on demand. This hydrogen storage strategy leverages a reversible process where CO_2_ is initially captured by amines under hydrogenation conditions with an appropriate catalyst, yielding formamides. When hydrogen is required, the system operates in reverse: the formamide first undergoes hydrolysis to produce formic acid and the parent amine, followed by formic acid dehydrogenation to generate H_2_ and CO_2_. The released CO_2_ is then recaptured by the amine, completing the sustainable cycle.

**Scheme 3:**
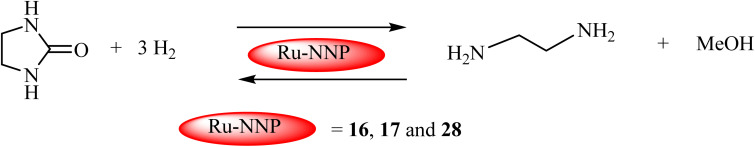
Hydrogen storage in methanol *via* ethylene urea using NNP-based Ru catalysts.^84^*Adapted with permission from ref. 84 from Wiley-VCH Verlag GmbH & Co. KGaA, Weinheim, Angew. Chem., 2019,****131****, 5159, Copyright 2019*.

**Fig. 6:**
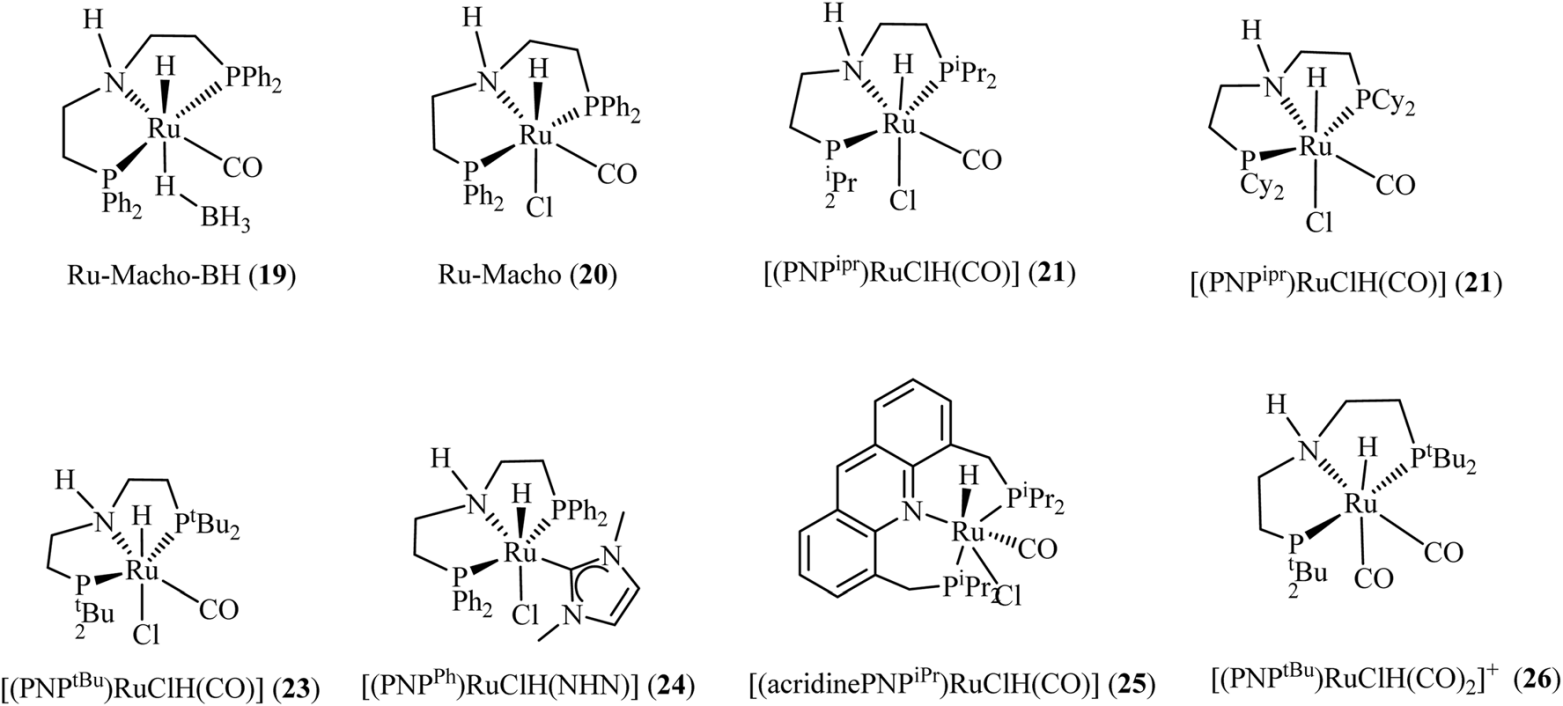
Example of phosphorus-donor catalysts used by Prakash *et al.*^81^ for catalyzing methanol synthesis from CO_2_. *Adapted with permission from**ref*. *81**from the American Chemical Society, J. Am. Chem. Soc*.*, 2019,****141****, 3160, Copyright 2019.*

**Fig. 7:**
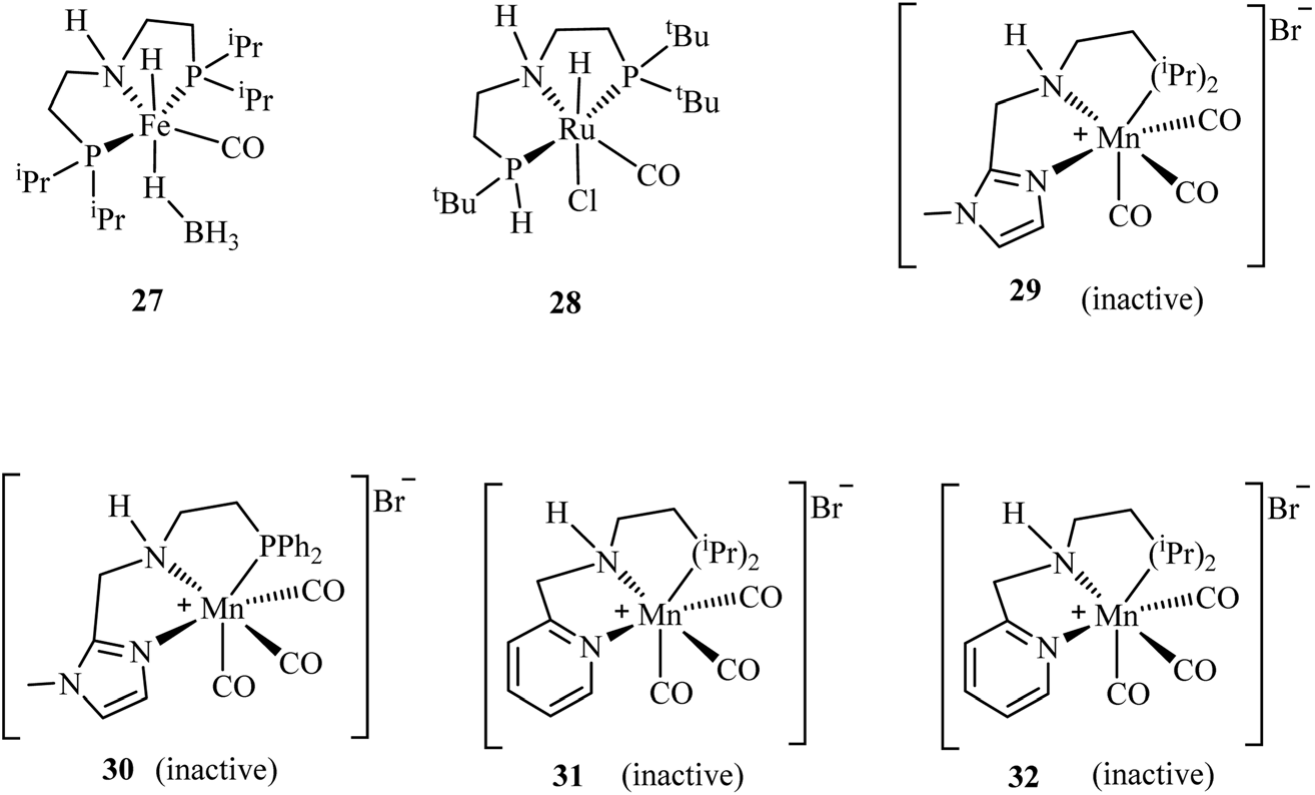
Examples of phosphorus-based catalysts explored for hydrogen release from methanol *via* amides.

Beller and coworkers explored the dehydrogenation of formamides using iron-based PNP-pincer complex 27 (Fig. 7) as non-noble metal catalysts, achieving >70% hydrogen yield.^83^ Remarkably, the PNP-Fe catalyst demonstrated exceptional stability, retaining >99% hydrogen selectivity even after 10 consecutive reaction cycles. This highlights the catalyst's potential for sustainable hydrogen production. Milstein's team achieved a breakthrough in methanol production by demonstrating the first successful catalytic hydrogenation of ethylene urea to methanol (Scheme 3).^84^ The system enables complete CO_2_ recycling, where the dehydrogenated byproduct can be reconverted to ethylene urea through reaction with ethylenediamine. This cyclic process was facilitated by Ru-pincer complexes 16, 17, and 28 (Fig. 6) featuring phosphorus-donor ligands. Under optimized hydrogenation conditions (1% catalyst loading, 1.0 mmol ethylene urea, 60 bar H_2_, 4.0 mmol ^*t*^BuOK in 2.0 mL dioxane at 170 °C for 7 days), all three catalysts achieved complete substrate conversion. Catalyst 16 demonstrated superior performance, yielding 81% methanol, the highest recorded in the study. While the current protocol requires elevated temperatures and extended reaction times, it establishes a proof-of-concept for a novel LOHC system. The reverse dehydrogenation process proved significantly more efficient, with catalyst 28 (0.01 mol%) completing the transformation in just 48 h at 150 °C. This step primarily generated molecular hydrogen, along with minor quantities of formamide derivatives, recycled ethylene urea, and trace CO in some cases. The marked difference in reaction times between hydrogenation and dehydrogenation suggests opportunities for catalyst optimization to improve the system's overall energy efficiency.

Liu and coworkers^85^ developed a hydrogen storage system utilizing phosphorus-based NNP and PNP manganese complexes 9, 11, and 29–32 (Fig. 7) to facilitate the reversible interconversion between methanol-diamine and diamide pairs. Under optimized reaction conditions (2 mol% catalyst 11, 4 mol% ^*t*^BuOK, 1 equivalent dimethylethylenediamine in 0.4 mL dioxane at 165 °C for 16 h), the system demonstrated exceptional selectivity for hydrogen production, achieving a 92% yield with minimal amine-derived byproducts. Comparative evaluation revealed stark performance differences among the catalysts: while complex 9 afforded a modest 48% hydrogen yield, the remaining four catalysts showed no detectable hydrogen production.^85^

**Scheme 4:**
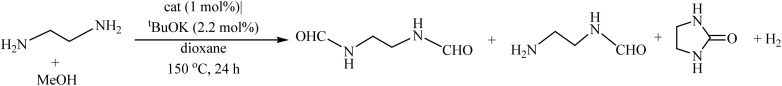
Catalytic hydrogen release from methanol *via* amides.^84^*Adapted with permission from ref. 84 from Wiley-VCH Verlag GmbH & Co. KGaA, Weinheim, Angew. Chem., 2019,****131****, 5159, Copyright 2019.*

Mechanistic studies suggest a dehydrogenation pathway where dimethylethylenediamine and methanol initially form diamide intermediates. Key reaction intermediates, including formaldehyde and monoamide derivatives (Scheme 4), function as sequential hydrogen carriers that undergo progressive dehydrogenation, ultimately releasing molecular hydrogen.^84^ This cascade transformation highlights the potential of phosphorus-based manganese catalysts for efficient hydrogen storage and release systems.

### 3.4 Phosphorus-mediated homogeneously catalyzed hydrogen evolution from water

**Fig. 8:**
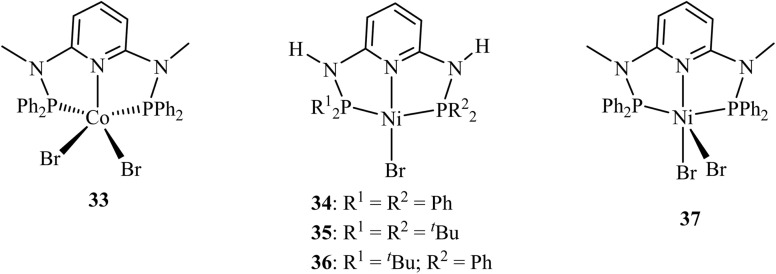
Co- and Ni-PN_3_P complexes used in hydrogen evolution reaction.

Water, the combustion product of hydrogen, represents the most desirable feedstock for hydrogen generation. Realizing hydrogen production *via* electro/photo-catalytic reduction of pure water, and/or seawater, remains a critical objective with profound implications for sustainable energy systems.^86^ Consequently, the pursuit of highly efficient homogeneous catalysts for hydrogen evolution has been a persistent focus in the field. Notably, certain phosphorus-based complexes have emerged among the most effective homogeneous systems for catalytic hydrogen generation from water. A representative example is the use of a CoPN_3_P complex (Fig. 8) for electrocatalytic hydrogen evolution from water in an acetonitrile medium. The system achieved an impressive 96% faradaic efficiency for H_2_ generation with complex 33. Using a catalyst concentration of 9.4 × 10^−4^ M, electrode surface area of 1.38 cm^2^, the rate of hydrogen formation from pure water reached 571 µmol.^86^

In a separate study involving the photocatalytic production of H_2_, Strabler *et al.*^87^ employed a Co diimine-dioxime molecular complex. They observed a doubling of the TON when a phosphane ligand was added to the reaction medium. Notably, the presence of the phosphane additive in the reaction medium proved critical for stabilizing the active species, hence significantly enhancing the catalytic performance, with the TON reaching 770.^87^

Electrocatalytic hydrogen evolution in acetonitrile/water mixtures has recently been achieved using air-stable NiPN_3_P pincer complexes (34 and 37), with water functioning as the proton source. TOFs reaching 160 s^−2^ and an 88% faradaic efficiency at −2.55 V (*vs.* Fc^+^/Fc) were recorded with systems under high water content.^88^ Subsequent investigations examined how variations in phosphorus substituents within the PN_3_P ligand framework (34–36) affect catalytic behaviour. A combined experimental and theoretical approach revealed that stepwise substitution of *tert*-butyl groups, electron-donating moieties by unsubstituted phenyl groups, and electron-withdrawing on one phosphorus atom introduced significant electronic alterations. These adjustments influenced the Ni(ii/i) and Ni(i/0) reduction potentials and modified the p*K*a of intermediate metal hydrides. Such modifications are critical because they directly govern reaction kinetics, catalytic rates, and the overpotentials necessary for efficient electrocatalytic hydrogen evolution.^89^

**Fig. 9:**
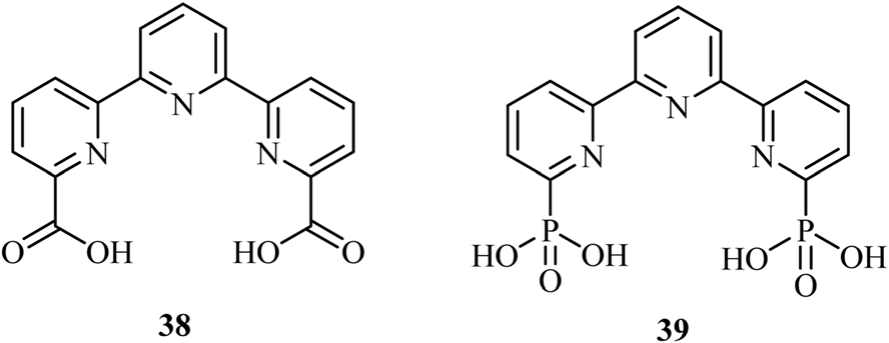
Representative carboxylate and phosphonate-based ligands in hydrogen evolution.

Additionally, various studies^90^ on water oxidation have revealed that introducing phosphonate or carboxylate groups (Fig. 9) into the secondary coordination sphere of ruthenium-based catalysts enhances their ability to act as proton acceptors. These groups facilitate proton-coupled electron transfer as well as promoting O–O bond formation.^91^ Notably, complexing Ru with 38 has demonstrated an impressive maximum turnover frequency (TOF_ma*x*_) of approximately 8000 s^−1^ at pH 7.0.^92^

**Scheme 5:**
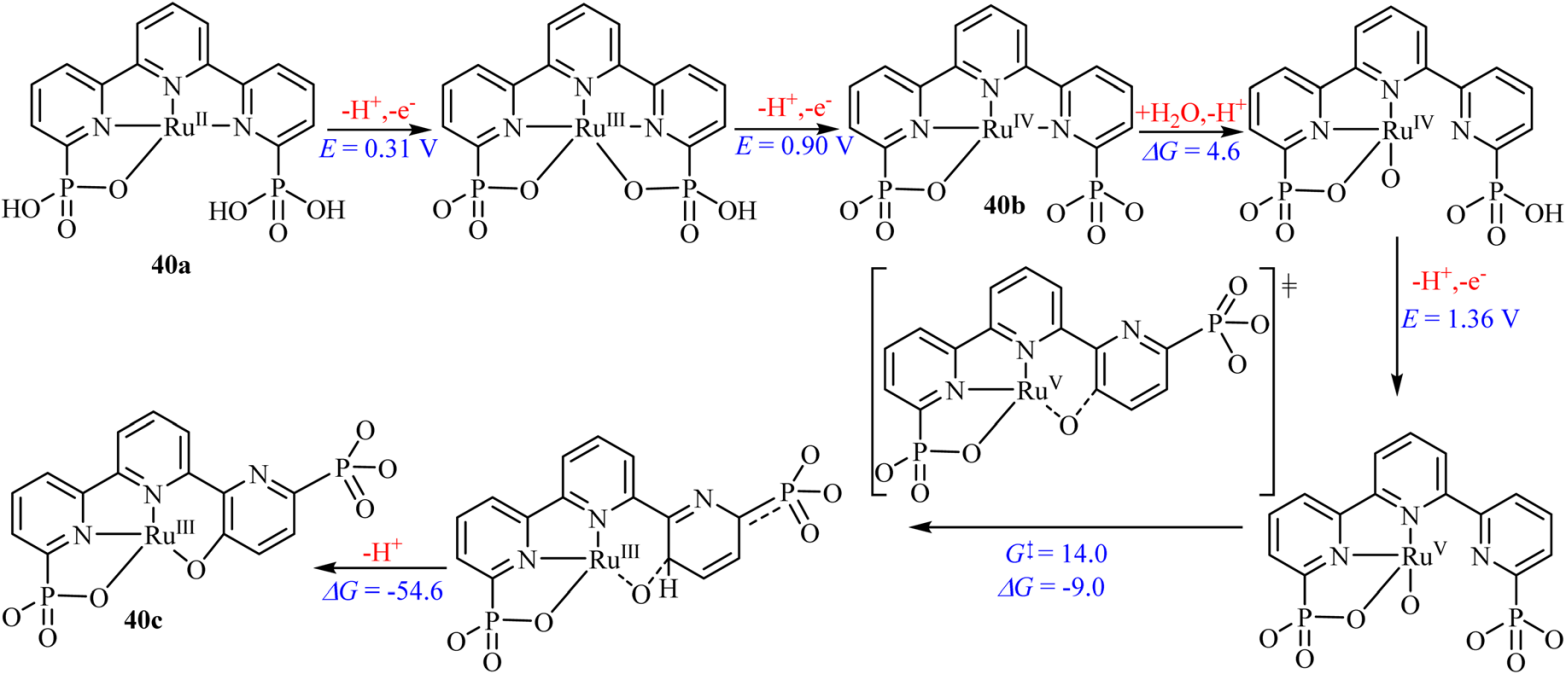
Proposed pathway for generating the active specie [Ru^III^(tPaO-κ-N^2^O_P_O_C_)(py)_2_]^2–^ (40c), from the precursor [Ru^II^(H_2_tPa-κ-N^3^O)(py)_2_](40a).^92^*Adapted with permission from ref. 92 from American Chemical Society, J. Am. Chem. Soc., 2020,****142****, 5068, Copyright 2020.*

However, replacing the carbonate groups in 38 with phosphonate (39) resulted in doubling the TOF_max_ values under similar conditions. Vereshchuk *et al.*^92^ attributed the superior performance to the role of the dangling phosphonate group, which provides a low-energy pathway for both generating the active species through oxygen insertion and intramolecular proton transfer from the coordinated water molecule during O–O bond formation, which is the rate-determining step in the water nucleophilic attack mechanism (Scheme 5).^92^ These studies illustrate the important role of phosphorus-based catalysts in redox catalysis.

### 3.5 Phosphorus-mediated homogeneously catalyzed hydrogen evolution from ammonia-borane

Among the various candidates for efficient hydrogen storage materials, AB has attracted considerable attention due to its exceptionally high hydrogen content (19.6 wt%), low cost, relative stability, low toxicity, and commercial availability.^93^ These advantageous properties make AB an attractive hydrogen carrier for applications in hydrogen-powered transportation systems and proton exchange membrane (PEM) fuel cells.^94,95^

**Fig. 10:**
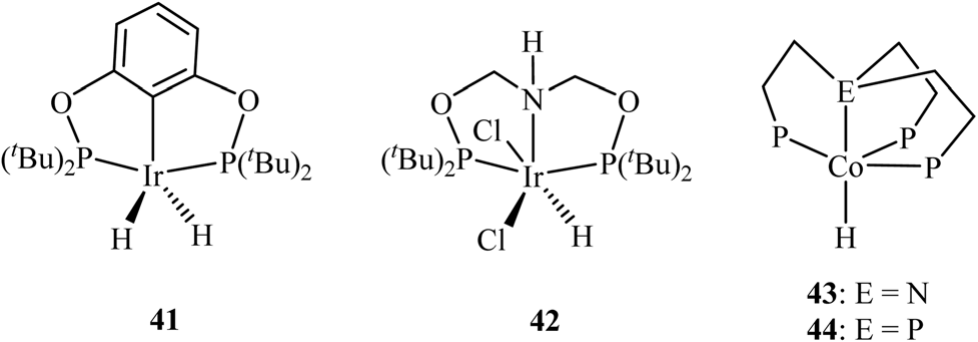
Selected phosphorus-based compounds for AB dehydrogenation.

The first example of homogeneous catalytic dehydrogenation of AB was reported in 2006 by Goldberg and co-workers, who demonstrated that the iridium pincer complex 41, (POCOP)Ir(H)_2_ efficiently catalyzes the dehydrogenation of AB at room temperature (Fig. 10). This system rapidly generated one equivalent of H_2_ along with the cyclic oligomer [NH_2_BH_2_]_5_ within 14 minutes.^96^ This seminal discovery stimulated extensive research over the past two decades aimed at developing phosphorus-containing homogeneous catalysts for AB dehydrogenation.

Subsequent advances were reported in 2010 by Graham *et al.*,^97^ who developed an efficient catalytic system for the solvolysis of AB using an Ir–PNP pincer complex 42 in a 1 : 1 ^*i*^PrOH/H_2_O solvent mixture. Under these conditions, the system produced 2.93 equivalents of H_2_ within 10 minutes and exhibited good catalyst recyclability over ten catalytic cycles.^97^ Conley and Williams^98^ also employed Shvo's catalyst in AB dehydrogenation to release two equivalents of hydrogen, giving borazine as a by-product. The reaction follows an outer-sphere pathway wherein hydride transfer from the metal to the boron centre of ammonia borane liberates dihydrogen and leads to borazine formation. Transition-metal complexes based on earth-abundant metals have also been investigated. For example, Todisco *et al.*^99^ reported tetradentate Co(i) complexes 43 and 44 for the dehydrogenation of AB in THF at 55 °C.^99^ Complex 43 generated two equivalents of H_2_ per equivalent of AB within 48 h. In contrast, complex 44 produced only one equivalent of H_2_ under identical conditions, highlighting the significant influence of ligand donor properties on catalytic performance.

### 3.6 Catalyst deactivation pathways and mechanistic insights

The long-term stability of phosphorus-based catalysts is critical for practical applications, yet deactivation mechanisms remain underexplored in many systems. A comprehensive understanding of deactivation pathways is essential for the rational design of more durable catalysts.^100^

In ruthenium PNP-catalysed CO_2_ hydrogenation to methanol, a key step in hydrogen storage *via* liquid organic hydrogen carriers, deactivation primarily occurs through the formation of biscarbonyl complexes.^81^ The accumulation of CO byproducts leads to the formation of [RuHPNP(CO)_2_]^+^ species, which exhibit reduced ligand dissociation tendencies and lower catalytic activity.^81^ This deactivation pathway is highly dependent on the steric and electronic properties of the phosphine ligands; catalysts with electron-donating, bulky substituents (*e.g.*, ^*i*^Pr, Cy, ^*t*^Bu) are particularly susceptible, while those with phenyl-substituted phosphines show greater resistance.^81^

Beyond biscarbonyl formation, catalyst deactivation in formic acid dehydrogenation has been attributed to the accumulation of reaction intermediates on active sites. Studies on Pd/AC catalysts for continuous hydrogen production from formic acid revealed that both reversible sorption of formate species and irreversible chemisorption of CO_2_ progressively block active sites, leading to declining catalytic activity over time.^101^ Additionally, the reduction of Pd^2+^ to Pd^0^ was identified as a contributing factor to deactivation in this system.^101^

For AB, a high-density hydrogen carrier, catalyst deactivation can result from reaction with free BH_3_ generated *via* aminoborane rearrangement.^102^ This deactivation pathway can be mitigated through borane trapping by simple amines, which prevents BH_3_ from coordinating to and poisoning the metal centre.^102^ Such strategies have enabled unprecedented turnover numbers in iron-catalysed AB dehydrogenation, highlighting the importance of understanding deactivation mechanisms for rational catalyst design.^102^ The formation of stable resting states represents another deactivation mechanism relevant to hydrogen release. For iridium POCOP pincer complexes in AB dehydrogenation, the Ir-H (η^2^-BH_4_) intermediate has been identified as a catalyst poison that prevents further activation of ammonia borane.^102^ Similarly, the observation of stable (^*t*^Bu)_2_PNP Co-H_2_O and (^*t*^Bu)_2_PNP Co-NH_3_ chelation products during AB hydrolysis suggests that careful control of reaction conditions is required to maintain catalytic activity.^102^

For phosphorus-based photocatalysts used in hydrogen evolution from water, deactivation primarily occurs *via* oxidation at the phosphorus surface, forming P_*x*_O_*y*_ species that degrade electronic properties and reduce catalytic activity.^30,103^ Upon prolonged exposure to ambient conditions or aqueous reaction media, the lone pair electrons on BP surfaces react with oxygen, leading to gradual corrosion through the formation of phosphoric acid.^103^ This instability has been identified as a major barrier to industrial implementation.^103^ Strategies to mitigate this include surface passivation, encapsulation in protective layers (*e.g.*, carbon or metal oxides), and the development of hybrid structures that stabilize BP against oxidation while maintaining catalytic function.^30,103^

For TMPs employed as electrocatalysts for hydrogen evolution, deactivation in acidic media can occur through metal dissolution.^104^ Studies on Co_2_P catalyst in HER revealed stochiometric compositions of both Co and P in acidic media, pointing to the dissolution of both Co and P.^105^ In contrast, alkaline media promoted the preferential dissolution of P, and *in situ* metal hydroxide formation, consequently decreasing the HER activity.^105^

Recent advances in doping modification have shown that element doping is an efficient way to greatly improve the activity and stability of TMPs.^52^ Through systematic doping, whether with metals, non-metals, or co-doping, the electronic structure of TMPs can be tuned to enhance both catalytic activity and resistance to deactivation.^52^ For black phosphorus quantum dots, nonmetallic heteroatom doping (*e.g.*, B, C, N, O) has been shown to improve stability while simultaneously enhancing hydrogen evolution reaction activity by lowering the HER barrier.^106^

## 4. Theoretical perspective

**Fig. 11:**
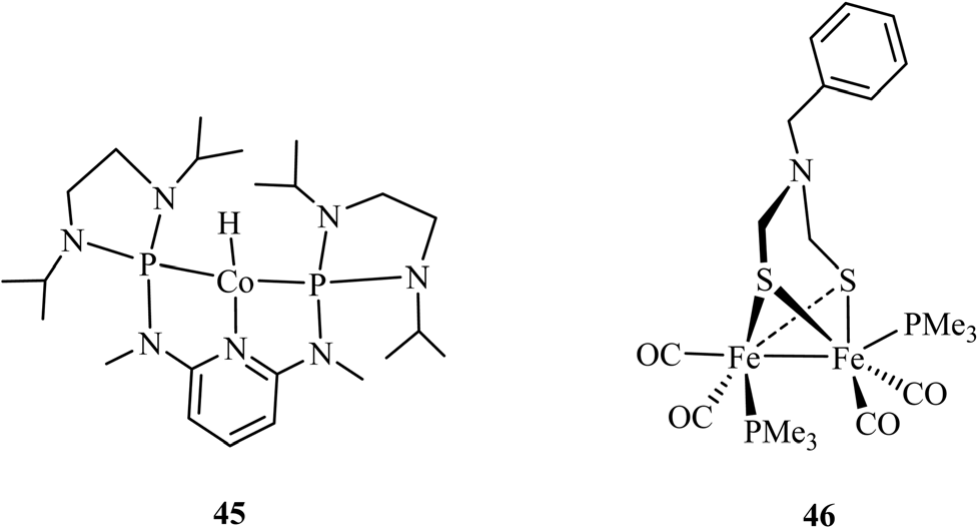
Phosphorus as an electronic tuner in molecular catalysts. 45: Cobalt (ii) PNP pincer complex for CO_2_ hydrosilylation, where phosphorus ligands modulate the hydride affinity (Δ*G*_H_^−^). 46: Diiron dithiolate [FeFe]-hydrogenase mimic with phosphine ligands, where phosphorus donors alter the redox potential and hydricity of the diiron centre. Both exemplify how phosphorus coordination governs key electronic descriptors to control catalytic activity.

The most significant advancement in rational catalyst design has been the marriage of computational chemistry with experimental validation.^107^ In their work on CO_2_ hydrosilylation, Cramer *et al.* employed hydride affinity (Δ*G*_H_^−^) as a universal descriptor to map the activity and selectivity of dozens of pincer catalysts.^108^ Hydride affinity is a computational descriptor that quantifies the thermodynamic tendency (given by the Gibbs free energy change, Δ*G*) of a metal complex to donate or accept a hydride ion (H^−^), serving as a predictive measure of its intrinsic reactivity in catalytic cycles involving hydrogen transfer. Their computational screening revealed how replacing nitrogen donor arms with phosphorus in pincer ligands (*e.g.*, moving from NNN to PNP frameworks) systematically tunes the Δ*G*_H_^−^, directly correlating with stronger metal-hydride bonds and altering the rate-determining steps across multi-cycle reaction networks. This theoretical model not only rationalized past catalytic behaviour but also successfully forecasted a new outcome. Specifically, it predicted that a modified cobalt PNP pincer complex (45) would achieve >80% yield of the challenging formaldehyde product (Fig. 11). Subsequent experimental tests validated this prediction, confirming the model's power.^108^ This study provides a blueprint for understanding the role of phosphorus in catalysis, demonstrating that its function is quantifiable through parameters like Δ*G*_H_^−^, which predicts how electron density donated by P-ligands influences the thermodynamic and kinetic landscape of hydride-involving cycles.

The predictive power of theory is equally pronounced in heterogeneous catalysis. For hydrogen evolution reaction (HER) catalysts like transition metal phosphides (TMPs, *e.g.*, Ni_2_P, CoP), experimental observations of high activity were initially intriguing. Theoretical studies provided the foundational “why”. Early DFT work established that the hydrogen adsorption free energy (Δ*G*_H*_) is the key activity descriptor for HER.^109^ Calculations showed that on Ni_2_P surfaces, P sites act not as inert spectators but as proton-acceptor bases that optimize Δ*G*_H*_ to near the thermoneutral ideal (∼0 eV), while neighboring metal sites facilitate H–H bond formation.^110^ This bifunctional mechanism, elucidated by theory, explains the superior performance of TMPs over pure metals and has guided the design of more complex ternary phosphides.^111^

In molecular catalysis for hydrogen storage and homogeneous H_2_ activation, phosphine ligands are ubiquitous. Their function extends beyond steric protection to precise electronic tuning. Theoretical studies on model systems, such as those relevant to [FeFe]-hydrogenase mimics, quantify how phosphine ligands alter the redox potentials and hydricities of metal centres.^112^ For instance, replacing a single P-donor in a diiron complex (46) can shift the potential for proton reduction by hundreds of millivolts and change the favourability of hydride donation.^113^ These computed parameters provide a quantitative language to explain experimental trends in catalytic rates and overpotentials across different phosphine ligand libraries.

A review that integrates these theoretical perspectives moves the field from empirical correlation to mechanistic prediction. A unified mechanistic principle emerging from these studies is that phosphorus acts as an electronic lever. By fine-tuning electron density at the metal centre, it controls fundamental energetic descriptors (Δ*G*_H_^−^, Δ*G*_H*_, hydricity, redox potential), thereby dictating the activity and selectivity of the catalyst. Consequently, the true potential of phosphorus in catalysis will be unlocked through a paradigm shift. This shift requires employing theoretical tools proactively to calculate descriptors and screen candidate structures before synthesis. This will transition research from slow, serendipitous discovery to rational, predictive design, dramatically accelerating the development of next-generation catalysts for hydrogen technology.^114^

**Scheme 6:**
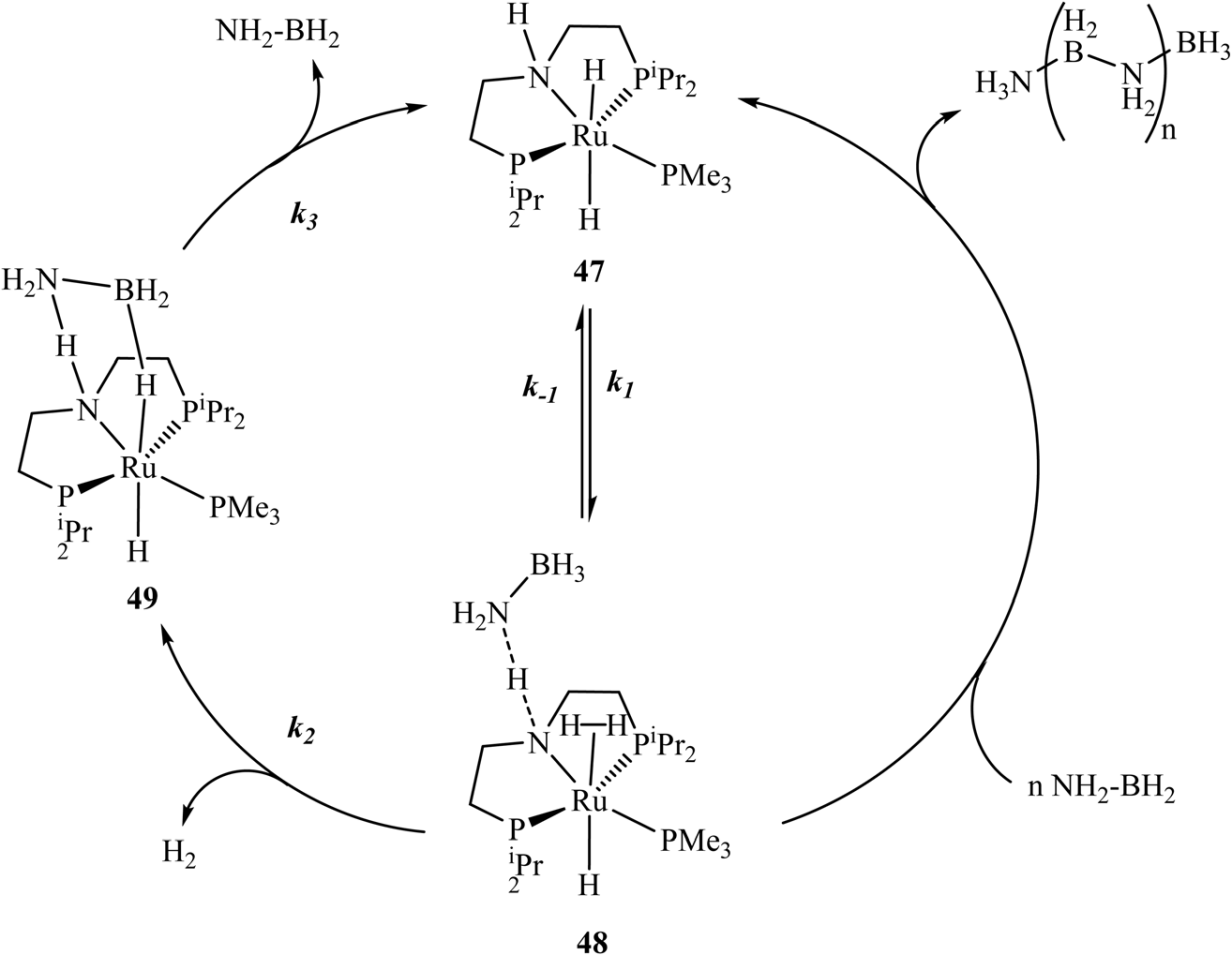
Plausible mechanism for dehydrocoupling of AB with Ru-PNP catalyst 47.^115^*Adapted with permission from ref. 115 from the American Chemical Society, J. Am. Chem. Soc.*, *2013,****135****, 13342*, *Copyright 2013*.

Marziale *et al.*^115^ performed extensive theoretical and experimental studies probing the mechanism of AB dehydrocoupling with phosphorus-based ruthenium catalysts using various approaches, including DFT calculations. Based on the findings, they proposed that for the complex 47, two related mechanistic cycles account for both dehydrogenation to aminoborane (H_2_N–BH_2_) and B–N coupling *via* metal-catalysed routes (Scheme 6). The left-hand catalytic cycle describes the dehydrogenation of AB, leading to the release of hydrogen gas and the formation of H_2_N–BH_2_. In contrast, the right–hand cycle outlines a metal-assisted pathway for oligomerization, where H_2_N–BH_2_ inserts into an N–H bond of the substrate. Computational studies indicate that this pathway, proceeding through a dihydrogen intermediate (48), has very low activation barriers, which accounts for the predominant formation of linear oligomers of the type H_3_N–(BH_2_–NH_2_)_n_–BH_3_. Additionally, B–N bond formation mediated by 47 is predicted by DFT calculations to occur with minimal energy barriers and is nearly thermoneutral, suggesting that the process is readily reversible.

Li's work^102^ on the mechanistic investigation of AB hydrolysis by various phosphorus-based compounds using DFT methods sheds more light on theory-driven rational design of efficient catalysts for HER. In the study, the behavior of AB towards Co, Ir, Ru, and Fe was probed *via* concerted, proton-transfer, and stepwise mechanisms. Importantly, the theoretical results align with the experimental findings. Furthermore, the analysis of various Ir-PNP complexes revealed that bulkier substituents on the P-donor favour higher activities following the order, (^*t*^Bu)_2_P > (^*i*^Pr)_2_P > (Ph)_2_P, *via* the concerted pathway.^102^

## 5. Toward sustainable phosphorus utilization in catalysis

The finite nature of phosphorus resources, with global phosphate reserves projected to face depletion pressures in the coming decades, necessitates a paradigm shift toward sustainable management practices throughout the catalyst lifecycle.^116^ Several complementary strategies have emerged to address this challenge as discussed in the following sections.

### 5.1 Catalyst immobilization and heterogenization

One of the most effective approaches for improving phosphorus sustainability is the immobilization of molecular catalysts on solid supports, enabling efficient recovery and reuse while minimizing phosphorus loss.^117^ Recent advances in phosphorus-enriched organic polymer supports have demonstrated exceptional promise in this regard. For example, Liu and co-workers developed cross-linked organic polymers (COPs) synthesized using (±)-2,2′-bis(diphenylphosphino)-1,1′-binaphthyl (BINAP) as monomers for use as supports for palladium catalysts.^117^ The resulting COP-BINAP-PdCl_2_ catalyst maintained high catalytic activity and structural stability even after five reuse cycles, with consistent Pd loading amounts.^117^ This approach effectively addresses challenges related to recycling and reusing homogeneous precious metal catalysts while simultaneously conserving the phosphorus content embedded within the phosphine ligand frameworks, offering a promising strategy to alleviate metal scarcity, reduce environmental pollution risks, and minimize phosphorus loss.

Additional immobilization strategies include covalent attachment to silica or polymer matrices, encapsulation in metal–organic frameworks (MOFs), incorporation into ionic liquid phases, and the use of magnetic nanoparticles for facile recovery.^48^ For transition metal phosphide catalysts, encapsulation with heteroatom-doped carbon shells has been shown to prevent active phase deactivation while facilitating magnetic recovery and reuse.^48^ Liu and co-workers comprehensively reviewed these strategies, emphasizing that the choice of support material and immobilization method must balance catalyst accessibility, stability, and recyclability.^48^

### 5.2 Design of durable catalytic systems

The development of catalytic systems with extended lifetimes directly reduces phosphorus consumption per unit of hydrogen produced. The manganese pincer complexes reported by Wei and Beller and co-workers, which retained over 90% activity over ten recycling cycles in CO_2_ hydrogenation, exemplify this approach.^66^ Specifically, the complex 14 (Fig. 4) was recycled ten times while maintaining over 90% yield in formic acid dehydrogenation, demonstrating exceptional robustness under harsh reaction conditions.^66^ Similarly, the Ru-Macho-BH catalyst (19) developed by Kar and Prakash and co-workers demonstrated stable performance over 10 days of continuous operation in methanol production from CO_2_, achieving a total turnover number of 9900.^81^

The importance of catalyst durability in phosphorus-based systems is further illustrated by the work of Amenuvor and co-workers, who developed hexanuclear Ru(ii)_4_^Zn(ii)_2_ complexes supported by diphenylphosphine ligands.^118^ These multimetallic catalysts, which feature phosphorus-rich coordination environments, were successfully recycled up to seven times in the hydrogenation of levulinic acid to γ-valerolactone using formic acid as the hydrogen source, a reaction directly relevant to hydrogen storage *via* liquid organic hydrogen carriers. The catalysts achieved turnover frequencies as high as 540 h^−1^ under mild, solvent-free conditions, demonstrating that robust phosphine-ligated frameworks can sustain multiple catalytic cycles without significant phosphorus loss.^118^

The exceptional stability of these systems is attributed to the robust coordination environment provided by phosphine ligands, which resist decomposition under a range of reaction conditions.^66,81,118^ In the case of the manganese pincer catalysts, the incorporation of electron-donating phosphine substituents (*e.g.*, ^*i*^Pr groups on the phosphorus centres) was found to enhance the stability of the metal-hydride intermediates, preventing off-pathway decomposition.^66^ These examples illustrate that rational ligand design, guided by mechanistic understanding, can yield catalysts capable of extended operation, thereby reducing the overall phosphorus demand for a given hydrogen production or storage application.

### 5.3 Phosphorus recovery from spent catalysts

Phosphorus recovery from spent catalysts represents a critical but currently underexplored opportunity. Drawing from broader phosphorus recovery research, hydrometallurgical processes including acid leaching and solvent extraction have been developed for recovering phosphorus from waste streams, though their application to catalyst recycling remains emerging.^116^ For example, struvite (MgNH_4_PO_4_·6H_2_O) precipitation has been demonstrated as an effective method for phosphorus recovery from process wastewaters, achieving 99% P-recovery with over 90% purity under optimized conditions (pH 9, 20 °C, equimolar input ratio of constituent ions).^119^ Widderich and co-workers demonstrated that such processes can be integrated into bio-based circular economy frameworks, where phosphorus mobilized from biomass is recovered and repurposed.^119^

Adapting such approaches for catalyst-specific waste streams, such as spent phosphine-ligated complexes or transition metal phosphide materials, could significantly improve phosphorus circularity.^116,119^ Key challenges include the selective extraction of phosphorus from mixed metal–ligand systems, the recovery of phosphorus in a form suitable for catalyst resynthesis, and the economic viability of recovery processes compared to primary phosphorus mining.^116^ Addressing these challenges will require collaboration between catalyst designers, separation scientists, and process engineers.

### 5.4 Quantitative metrics for phosphorus efficiency

A comparative assessment of phosphorus utilization efficiency across catalyst systems would enable more informed choices in catalyst selection and development. Following the principles established by Bligaard and Nørskov for catalyst benchmarking, we propose metrics such as Turnover Number per Phosphorus atom (TON/P) and Turnover Frequency per Phosphorus atom (TOF/P) as normalized measures of phosphorus efficiency.^120^ These metrics allow fair comparison between different catalytic platforms, for instance, comparing a molecular complex containing one phosphorus atom per metal centre against a heterogeneous phosphide material containing multiple phosphorus atoms per active site.

For a catalyst of the formula ML_*n*_P_*x*_, where M is the metal, L is an ancillary ligand, and P_*x*_ represents the number of phosphorus atoms per catalytic unit, TON/P is defined as:
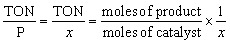


Similarly, TOF/P normalizes the turnover frequency by the phosphorus content:
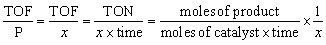


These metrics enable direct comparison of phosphorus efficiency across catalysts with different stoichiometries. For example, a molecular complex with TON = 10 000 and one phosphorus atom per metal centre (*x* = 1) yields TON/P = 10 000, whereas a transition metal phosphide with TON = 50 000 but containing five phosphorus atoms per active site (*x* = 5) yields TON/P = 10 000 as well, indicating equivalent phosphorus utilization efficiency despite differing absolute TON values.

When combined with life-cycle assessment approaches that account for phosphorus mining, processing, catalyst synthesis, and end-of-life recovery, these metrics would help guide the selection of phosphorus-based catalysts toward the most sustainable options.^116,119^ The catalysis community is therefore encouraged to adopt such metrics in reporting, facilitating cross-system comparisons, and incentivizing the development of phosphorus-efficient catalysts.

### 5.5 Elemental phosphorus from sustainable sources

The sustainability of phosphorus-based catalysis ultimately depends on the source of elemental phosphorus used in catalyst synthesis. Current industrial production of white phosphorus, the precursor for most phosphine ligands and phosphorus allotropes, is energy-intensive, requiring the carbothermal reduction of phosphate rock at temperatures exceeding 1500 °C.^116^ This process carries a significant carbon footprint and generates substantial waste by-products.^116^

Several alternative routes have emerged to address these challenges. One promising approach is the electrochemical reduction of phosphate in molten salts, which enables direct extraction of white phosphorus from phosphate rock using clean electricity at temperatures approximately 700 °C lower than conventional methods. Surendranath and co-workers demonstrated that increasing the Lux acidity of molten electrolytes promotes the reductive cleavage of strong P–O bonds, achieving 95% faradaic efficiency for the 5-electron (5e^−^) reduction of trimetaphosphate to P_4_.^121^

An orthogonal strategy avoids white phosphorus entirely through the direct conversion of phosphate to phosphorus-containing functional materials. Cummins and co-workers showed that several phosphorus fine chemicals can be accessed by treating phosphoric acid with trichlorosilane, bypassing the energy-intensive P_4_ intermediate.^122^ Weigand and colleagues subsequently developed a remarkably simple two-step process that directly converts primary and secondary phosphate sources into phosphorus-containing chemicals without using hazardous white phosphorus intermediates.^123^ The DFG-funded “Blueprint for a Modern Sustainable Phosphorus Chemistry” project further advances this approach by targeting redox-neutral synthesis of P(v) platform chemicals directly from recycled phosphate resources.^124^

A third approach leverages biomass-derived reductants as a renewable alternative to fossil-derived carbon coke. Wu and co-workers reviewed microbial digestion technology for phosphoric acid preparation, which offers mild operating conditions and minimal environmental pollution risks.^125^ Elser and colleagues highlighted the potential of bio-based phosphorus adsorption and transformation strategies, including the use of phosphate-solubilizing bacteria to enhance phosphorus availability from agricultural residues.^126^ These biotechnological approaches align with circular economy principles by recovering phosphorus from waste streams while reducing reliance on mined phosphate rock.^126^

Future research should prioritize the development of sustainable preparation methods for phosphorus allotropes and phosphine ligands, minimizing the carbon footprint of phosphorus catalyst production while maintaining the purity and functionality required for high-performance catalysis.

## 6. Conclusion and future directions

This review has systematically detailed the indispensable and multifaceted role of phosphorus in advancing hydrogen technology, spanning from production to storage. The versatility of phosphorus, from its solid-state allotropes (black, red, and violet phosphorus) to transition metal phosphides, high-entropy phosphides, and molecular coordination in ligands, establishes it as a critical enabler for both heterogeneous and homogeneous catalytic processes central to the hydrogen economy. Beyond documenting empirical successes, this review has established that a deeper, predictive understanding of phosphorus's role emerges from computational descriptors. Key parameters such as hydride affinity (Δ*G*_H_^−^) in molecular systems and hydrogen adsorption free energy (Δ*G*_H*_) on solid surfaces provide a quantitative link between the electronic influence of phosphorus and observed catalytic activity and selectivity. Mechanistic studies on ammonia borane dehydrocoupling, including DFT-based investigations of Ru-PNP catalysts, have further elucidated how phosphorus coordination environments dictate reaction pathways, whether simple hydrogen release or oligomer formation. This theoretical framework transforms phosphorus from a compositional element into a designable tuning parameter.

In heterogeneous photocatalysis, black phosphorus (BP) and its derivatives have emerged as exceptional materials, not merely as standalone catalysts but more powerfully as synergistic components in hybrid systems. The remarkable hydrogen evolution rates achieved by BP-based composites such as FLP/ZCS (9326 µmol h^−1^ g^−1^) and BP/Bi_2_WO_6_ (21 042 µmol h^−1^ g^−1^) underscore how BP's tunable bandgap and high charge carrier mobility can dramatically enhance the performance of metal sulfides and oxides. Beyond BP, red phosphorus and violet phosphorus quantum dots have demonstrated remarkable hydrogen evolution rates, with VPQD achieving 3325.1 mmol h^−1^ g^−1^. Transition metal phosphides such as Ni_2_P, CoP, and FeP have proven effective as cocatalysts, leveraging the cooperative interaction between proton-accepting P sites and electron-rich metal centers. Furthermore, high-entropy phosphide materials represent an emerging frontier, where the combination of five or more metals in a single phosphide phase creates unique electronic states optimally tuned for substrate adsorption and activation. The key finding is that the structural processing of phosphorus (*e.g.*, exfoliation to phosphorene, ball-milling) is a decisive factor in unlocking its full photocatalytic potential.

In homogeneous catalysis, phosphorus-based ligands are the cornerstone of high-performance molecular systems for hydrogen storage and release. The review has highlighted their dominance in reversible LOHC cycles, particularly for the CO_2_/formic acid and CO_2_/methanol couples. The inclusion of ammonia borane dehydrogenation catalysis further expands the scope, with iridium, ruthenium, and cobalt PNP complexes demonstrating efficient hydrogen release under mild conditions. The exceptional activities of complexes like *cis*-[Ru (DPPM)_2_Cl_2_] (TOF = 180 000 h^−1^) and Mn/Fe-pincer catalysts (TONs up to 3 500 000) demonstrate that the electronic and steric properties of phosphorus ligands are crucial for stabilizing active metal centers, facilitating key hydride transfer steps, and preventing catalyst deactivation. Understanding deactivation pathways, such as biscarbonyl formation, phosphine oxidation, metal dissolution, and surface oxidation, has emerged as essential for designing more durable catalysts. Furthermore, the successful application of non-precious metal (Mn, Fe, Co, Ni) complexes bearing phosphorus ligands marks a critical step toward cost-effective and sustainable catalytic systems. It is important to note that not all phosphorus-based catalysts exhibit exceptional performance. Several examples included in this review demonstrate modest activity, underscoring that phosphorus incorporation alone is insufficient without careful optimization of ligand electronics, sterics, and reaction conditions. Nevertheless, the sheer frequency with which phosphorus-containing systems appear in the literature (from black phosphorus photocatalysts to phosphine-ligated molecular complexes) affirms the central and growing role of phosphorus in catalyst design for hydrogen technology.

Despite these promising advances, several challenges and opportunities will shape future research directions. The escalating demand for phosphorus in energy technologies necessitates a paradigm shift toward sustainable management and this must be a pressing priority. Several strategies have been outlined (i) catalyst immobilization on solid supports (*e.g.*, COP-BINAP-PdCl_2_): to enable recovery and reuse; (ii) design of durable catalytic systems, exemplified by Mn pincer complexes recycled ten times and Ru-Macho-BH operating continuously for 10 days; (iii) phosphorus recovery from spent catalysts *via* hydrometallurgical processes such as struvite precipitation; (iv) quantitative metrics including TON/P and TOF/P to normalize phosphorus efficiency across different catalyst platforms; and (v) development of sustainable elemental phosphorus sources through electrochemical reduction of phosphate, direct conversion without P_4_ intermediates, and biomass-derived reductants.

The profound influence of phosphorus substituents on catalytic activity, which is evident in the stark performance differences between catalysts like 13–15 and the inactivity of 12, calls for deeper mechanistic studies. A more precise understanding of how ligand structure affects reaction kinetics and intermediate stability will enable the rational design of next-generation catalysts. For heterogeneous systems, efforts must focus on improving their long-term stability against oxidation (particularly for BP), developing scalable, cost-effective synthesis methods for high-entropy phosphides, and expanding the use of phosphorus-doped supports for AB hydrolysis. For homogeneous systems, simplifying catalyst synthesis and immobilizing molecular complexes on supports for easier separation and reuse are key goals. Additionally, the integration of theoretical descriptors into high-throughput screening workflows promises to accelerate catalyst discovery.

Future work should explore the integration of these advanced phosphorus-based catalysts into practical, large-scale hydrogen production and storage systems, assessing their performance under real-world conditions. Particular attention should be paid to the scalability of sustainable phosphorus sources and the economic viability of phosphorus recovery processes.

In conclusion, phosphorus is far more than a mere component; it is a pivotal element whose unique chemistry is driving innovation across the hydrogen value chain. From black phosphorus photocatalysts and high-entropy phosphides to phosphorus-ligated complexes for CO_2_ hydrogenation, methanol synthesis, formic acid dehydrogenation, ammonia borane hydrolysis, and water splitting, along with sustainable phosphorus management strategies, the breadth of phosphorus chemistry continues to expand. By leveraging the insights summarized in this review and addressing the associated challenges, the scientific community can harness the full potential of phosphorus to build an efficient and sustainable hydrogen economy.

## Author contributions

Gershon Amenuvor – conceptualization, writing original draft, review and editing. Juliana Mana Edor – writing, review and editing. Phillimon Modisha: supervision, review and editing. Dmitri Bessarabov – supervision and resources. Banothile C.E. Makhubela – resources and review.

## Conflicts of interest

There are no conflicts of interest to declare.

## Data Availability

No new data were created or analysed in this study. Data sharing is not applicable to this article.

## References

[cit1] Li R., Li Y., Yang P., Wang D., Xu H., Wang B., Meng F., Zhang J., An M. (2021). J. Energy Chem..

[cit2] Fang Y., Wang S., Lin G., Wang X., Huang F. (2021). Chem. Commun..

[cit3] Kim J.-C., Kim J., Park J. C., Ahn S. H., Kim D.-W. (2021). Chem. Eng. J..

[cit4] Peng Y., Liao Y., Ye D., Meng Z., Wang R., Zhao S., Tian T., Tang H. (2022). Nanomaterials.

[cit5] Tosti S., Basile A. (2003). Chem. Eng. J..

[cit6] Gross M. (2017). Curr. Biol..

[cit7] Neset T. S. S., Cordell D. (2012). J. Sci. Food Agric..

[cit8] Baveye P. C. (2015). Rev. Bras. Ciência do Solo.

[cit9] Alewell C., Ringeval B., Ballabio C., Robinson D. A., Panagos P., Borrelli P. (2020). Nat. Commun..

[cit10] Guo R., Lai X., Huang J., Du X., Yan Y., Sun Y., Zou G., Xiong J. (2018). ChemElectroChem.

[cit11] Kucernak A. R., Sundaram V. N. N. (2014). J. Mater. Chem. A.

[cit12] Yan J., Kong L., Ji Y., Li Y., White J., Liu S., Han X., Lee S.-T., Ma T. (2018). Commun. Chem..

[cit13] Chaudhary V., Neugebauer P., Mounkachi O., Lahbabi S., El Fatimy A. (2022). 2D Mater..

[cit14] Zhang W., Yao B., Yang H., Li X., Qiu L., Li S. (2024). Coatings.

[cit15] Castellanos-Gomez A. (2015). J. Phys. Chem. Lett..

[cit16] Sa B., Li Y.-L., Qi J., Ahuja R., Sun Z. (2014). J. Phys. Chem. C.

[cit17] Bridgman P. (1914). J. Am. Chem. Soc..

[cit18] Ozawa A., Yamamoto M., Tanabe T., Hosokawa S., Yoshida T. (2020). J. Mater. Chem. A.

[cit19] Tiouitchi G., Ali M. A., Benyoussef A., Hamedoun M., Lachgar A., Benaissa M., Kara A., Ennaoui A., Mahmoud A., Boschini F. (2019). Mater. Lett..

[cit20] Kitada S., Shimizu N., Hossain M. Z. (2020). ACS Omega.

[cit21] Smith J. B., Hagaman D., Ji H.-F. (2016). Nanotechnology.

[cit22] Guan L., Xing B., Niu X., Wang D., Yu Y., Zhang S., Yan X., Wang Y., Sha J. (2018). Chem. Commun..

[cit23] Sultana N., Degg A., Upadhyaya S., Nilges T., Sarma N. S. (2022). Mater. Adv..

[cit24] Cunningham G., Lotya M., Cucinotta C. S., Sanvito S., Bergin S. D., Menzel R., Shaffer M. S., Coleman J. N. (2012). ACS Nano.

[cit25] Ran J., Wang X., Zhu B., Qiao S.-Z. (2017). Chem. Commun..

[cit26] Yuan Y.-J., Wang P., Li Z., Wu Y., Bai W., Su Y., Guan J., Wu S., Zhong J., Yu Z.-T. (2019). Appl. Catal. B Environ..

[cit27] Zhu M., Zhai C., Fujitsuka M., Majima T. (2018). Appl. Catal. B Environ..

[cit28] Hu J., Chen D., Mo Z., Li N., Xu Q., Li H., He J., Xu H., Lu J. (2019). Angew. Chem., Int. Ed..

[cit29] He W., Dong H., Zhao P., Huang Y., Wang B., Gan Z., Lu H., Zhang R., Sui L., Dong L., Yu L. (2021). J. Alloys Compd..

[cit30] Zhu X., Zhang T., Sun Z., Chen H., Guan J., Chen X., Ji H., Du P., Yang S. (2017). Adv. Mater..

[cit31] Xu Y., Guo X., Song Z., Guan C., Yang C., Li T., Lu H., An C., Zhu Y. (2025). Carbon Neutralization.

[cit32] Wang F., Ng W. K. H., Yu J. C., Zhu H., Li C., Zhang L., Liu Z., Li Q. (2012). Appl. Catal. B Environ..

[cit33] Hu Z., Yuan L., Liu Z., Shen Z., Yu J. C. (2016). Angew. Chem..

[cit34] Wang X., Maeda K., Thomas A., Takanabe K., Xin G., Carlsson J. M., Domen K., Antonietti M. (2009). Nat. Mater..

[cit35] Li S., Ng Y. H., Zhu R., Lv S., Wu C., Liu Y., Jin L., Deng J., Dai H. (2021). Appl. Catal. B Environ..

[cit36] Wang X., Xu C., Wang Z., Wang Y., Zhao X., Zhang J., Ma M., Guo Q., Zhang F. (2023). J. Mater. Chem. A.

[cit37] Yang J., Wang D., Han H., Li C. (2013). Accounts Chem. Res..

[cit38] Meng A., Zhang L., Cheng B., Yu J. (2019). Adv. Mater..

[cit39] Liu P., Rodriguez J. A. (2005). J. Am. Chem. Soc..

[cit40] Cao S., Chen Y., Wang C., He P., Fu W. (2014). Chem. Commun..

[cit41] Shi Y., Zhang B. (2016). Chem. Soc. Rev..

[cit42] Cao S., Wang C., Fu W., Chen Y. (2017). ChemSusChem.

[cit43] Tan P., Zhu A., Liu Y., Ma Y., Liu W., Cui H., Pan J. (2018). Inorg. Chem. Front..

[cit44] Liu X., Zhao Y., Yang X., Liu Q., Yu X., Li Y., Tang H., Zhang T. (2020). Appl. Catal., B.

[cit45] Li Y., Li Y., Yang C., Yu C., Gan L. (2023). Appl. Surf. Sci..

[cit46] Zhuang C., Zhang A., Zhang Y., Zhu H., Zhang W., Shan P., Xu P., Jin Z., Huang H., Li S. (2025). Coord. Chem. Rev..

[cit47] Xiao P., Sk M. A., Thia L., Ge X., Lim R. J., Wang J., Lim K. H., Wang X. (2014). Energy Environ. Sci..

[cit48] Liu T., Chen C., Liu S., Chen Z., Pu Z., Huang Q., Zhang L., Enizi A., Nafady A., Sun S., Zhang G. (2024). Coord. Chem. Rev..

[cit49] Miracle D. B., Senkov O. N. (2017). Acta Mater..

[cit50] Zhao X., Sun W., Liu X., Lu Z., Chen K., Gao J., Chen J., Zhang H., Wen Z. (2025). Adv. Energy Mater..

[cit51] Jing C., Hong L., Li B., Wang Y., Zhang F., Huang H., Jiang Q., Tang J. (2024). Mol. Catal..

[cit52] Wang Z., Meng C., Yu R. (2022). Chem. J. Chinese Universities.

[cit53] Zhou H., Yu F., Zhu Q., Sun J., Qin F., Yu L., Bao J., Yu Y., Chen S., Ren Z. (2018). Energy Environ. Sci..

[cit54] Li Y., Gao X., Iv X., Duan Y., Sui D., Chang W., Yang Y. (2024). ChemCatChem.

[cit55] Liu H., Xu C., Lu R., Wang Q., Wu J., Wang Y., Zhang Y., Sun T., Fan G. (2019). Int. J. Hydrogen Energy.

[cit56] Wan C., Li G., Wang J., Xu L., Cheng D., Chen F., Asakura Y., Kang Y., Yamauchi Y. (2023). Angew. Chem., Int. Ed..

[cit57] Yao Q., Zhu F., Long J., Huo J., Huang M., Lu Z.-H. (2025). Chem. Eng. J..

[cit58] Kamiya H., Kato K., Xin Y., Xu Y., Shirai T. (2024). Catal. Lett..

[cit59] Edor J. M., Amenuvor G., Modisha P., Bessarabov D. (2026). React. Chem. Eng..

[cit60] Graf E., Leitner W. (1992). J. Chem. Soc., Chem. Commun..

[cit61] Gassner F., Leitner W. (1993). J. Chem. Soc., Chem. Commun..

[cit62] Inoue Y., Izumida H., Sasaki Y., Hashimoto H. (1976). Chem. Lett..

[cit63] Inoue Y., Sasaki Y., Hashimoto H. (1975). J. Chem. Soc., Chem. Commun..

[cit64] Scott M., Blas Molinos B., Westhues C., Franciò G., Leitner W. (2017). ChemSusChem.

[cit65] Sordakis K., Tang C., Vogt L. K., Junge H., Dyson P. J., Beller M., Laurenczy G. (2018). Chem. Rev..

[cit66] Wei D., Sang R., Sponholz P., Junge H., Beller M. (2022). Nat. Energy.

[cit67] Jessop P. G., Hsiao Y., Ikariya T., Noyori R. (1996). J. Am. Chem. Soc..

[cit68] Munshi P., Main A. D., Linehan J. C., Tai C.-C., Jessop P. G. (2002). J. Am. Chem. Soc..

[cit69] Elek J., Nádasdi L., Papp G., Laurenczy G., Joó F. (2003). Appl. Catal., A.

[cit70] Filonenko G. A., Van Putten R., Schulpen E. N., Hensen E. J., Pidko E. A. (2014). ChemCatChem.

[cit71] Huff C. A., Sanford M. S. (2013). ACS Catal..

[cit72] Tanaka R., Yamashita M., Nozaki K. (2009). J. Am. Chem. Soc..

[cit73] Schmeier T. J., Dobereiner G. E., Crabtree R. H., Hazari N. (2011). J. Am. Chem. Soc..

[cit74] Bertini F., Gorgas N., Stöger B., Peruzzini M., Veiros L. F., Kirchner K., Gonsalvi L. (2016). ACS Catal..

[cit75] Zhang Y., MacIntosh A. D., Wong J. L., Bielinski E. A., Williard P. G., Mercado B. Q., Hazari N., Bernskoetter W. H. (2015). Chem. Sci..

[cit76] Jeletic M. S., Mock M. T., Appel A. M., Linehan J. C. (2013). J. Am. Chem. Soc..

[cit77] Spentzos A. Z., Barnes C. L., Bernskoetter W. H. (2016). Inorg. Chem..

[cit78] Sen R., Goeppert A., Surya Prakash G. (2022). Angew. Chem., Int. Ed..

[cit79] Kar S., Goeppert A., Prakash G. S. (2019). Acc. Chem. Res..

[cit80] Khusnutdinova J. R., Garg J. A., Milstein D. (2015). ACS Catal..

[cit81] Kar S., Sen R., Kothandaraman J., Goeppert A., Chowdhury R., Munoz S. B., Haiges R., Prakash G. S. (2019). J. Am. Chem. Soc..

[cit82] Sen R., Koch C. J., Goeppert A., Prakash G. S. (2020). ChemSusChem.

[cit83] Wei D., Shi X., Junge H., Du C., Beller M. (2023). Nat. Commun..

[cit84] Xie Y., Hu P., Ben-David Y., Milstein D. (2019). Angew. Chem..

[cit85] Shao Z., Li Y., Liu C., Ai W., Luo S.-P., Liu Q. (2020). Nat. Commun..

[cit86] Rao G. K., Pell W., Gabidullin B., Korobkov I., Richeson D. (2017). Chem. Eur. J..

[cit87] Strabler C. M., Prock J., Viertl W., Pehn R., Weninger A., Kopacka H., Obendorf D., Brüggeller P. (2016). Mater. Today Proc..

[cit88] Norouziyanlakvan S., Rao G. K., Ovens J., Gabidullin B., Richeson D. (2021). Chem. Eur. J..

[cit89] Chatterjee S., Dutta I., Dereli B., Chakraborty P., Peramaiah K., Gupta N., Cavallo L., Huang K. W. (2024). Chem.–Asian J..

[cit90] Xiong H., Yang J., Li J., Cai Y., Zhang F., Chen J.-Y., Liao R.-Z., Sun L., Zhang B. (2025). ACS Catal..

[cit91] Matheu R., Z Ertem M., Benet-Buchholz J., Coronado E., Batista V. S., Sala X., Llobet A. (2015). J. Am. Chem. Soc..

[cit92] Vereshchuk N., Matheu R., Benet-Buchholz J., Pipelier M., Lebreton J., Dubreuil D., Tessier A., Gimbert-Suriñach C., Ertem M. Z., Llobet A. (2020). J. Am. Chem. Soc..

[cit93] Liu Q., Ran We., Bao W., Li Yu. (2025). Energies.

[cit94] Staubitz A., Robertson A. P. M., Manners I. (2010). Chem. Rev..

[cit95] Mondal B., Mohanty A., Daw P. (2026). Dalton Trans..

[cit96] Denney M. C., Pons V., Hebden T. J., Heinekey D. M., Goldberg K. I. (2006). J. Am. Chem. Soc..

[cit97] Graham T. W., Tsang C.-W., Chen X., Guo R., Jia W., Lu S.-M., Sui-Seng C., Ewart C. B., Lough A., Amoroso D., Abdur-Rashid K. (2010). Angew. Chem., Int. Ed..

[cit98] Conley B. L., Williams T. J. (2010). Chem. Commun..

[cit99] Todisco S., Luconi L., Giambastiani G., Rossin A., Peruzzini M., Golub I. E., Filippov O. A., Belkova N. V., Shubina E. S. (2017). Inorg. Chem..

[cit100] Peng C., Zhang Y., Wang Y., Liu W., Yang Y. (2023). Int. J. Hydrogen Energy.

[cit101] Martin C., Quintanilla A., Casas J. A. (2024). Catal. Today.

[cit102] Li Y., Tsang C.-W., Chan E. M. H., Wong E. Y. C., Ho D. C. K., Lu X.-Y., Liang C. (2020). Catalysts.

[cit103] Kuntz K. L., Wells R. A., Hu J., Yang T., Dong B., Guo H., Woomer A. H., Druffel D. L., Alabanza A., Tománek D., Warren S. C. (2017). ACS Appl. Mater. Interfaces.

[cit104] Du L., Zheng W. (2024). APL Energy.

[cit105] Zhang Y., Gao L., Hensen E. J. M., Hofmann J. P. (2018). ACS Energy Lett..

[cit106] Xu S., Feng J., Wang X., Sun Q., Dong H., Yu L., Dong L. (2025). Int. J. Hydrogen Energy.

[cit107] Wodrich M. D., Sawatlon B., Busch M., Corminboeuf C. (2021). Acc. Chem. Res..

[cit108] Cramer H. H., Das S., Wodrich M. D., Corminboeuf C., Werlé C., Leitner W. (2023). Chem. Sci..

[cit109] Nørskov J. K., Bligaard T., Logadottir A., Kitchin J. R., Chen J. G., Pandelov S., Stimming U. (2005). J. Electrochem. Soc..

[cit110] Liu P., Rodriguez J. A. (2005). J. Am. Chem. Soc..

[cit111] Popczun E. J., McKone J. R., Read C. G., Biacchi A. J., Wiltrout A. M., Lewis N. S., Schaak R. E. (2013). J. Am. Chem. Soc..

[cit112] Gloaguen F., Rauchfuss T. B. (2009). Chem. Soc. Rev..

[cit113] Schwartz L., Eilers G., Eriksson L., Gogoll A., Lomoth R., Ott S. (2006). Chem. Commun..

[cit114] Das S., Turnell-Ritson R. C., Dyson P. J., Corminboeuf C. (2022). Angew. Chem., Int. Ed..

[cit115] Marziale A. N., Friedrich A., Klopsch I., Drees M., Celinski V. R., auf der Günne J. S., Schneider S. (2013). J. Am. Chem. Soc..

[cit116] Cordell D., White S. (2011). Sustainability.

[cit117] Liu X., Han X., Cai J., Wang Q., Liu L., Duan X.-H., Hu M. (2025). Green Chem..

[cit118] Amenuvor G., Darkwa J., Makhubela B. C. E. (2018). Catal. Sci. Technol..

[cit119] Widderich N. , From Biomass to Bioeconomy: Engineering Biocatalytic Phosphorus Mobilization from Plant Residues Prior to Animal Feeding, PhD. Dissertation, Technische Universität Hamburg, Hamburg, Germany, 2025

[cit120] Bligaard T., Nørskov J. K. (2007). Electrochim. Acta.

[cit121] Melville J. L., Licini A. J., Surendranath Y. (2023). ACS Cent. Sci..

[cit122] Geeson M. B., Cummins C. C. (2018). Science.

[cit123] Schneider T., Schwedtmann K., Fidelius J., Weigand J. J. (2023). Nat. Synth..

[cit124] Deutsche Forschungsgemeinschaft (DFG) , Blueprint for a modern sustainable phosphorus chemistry, Project Number 524609036. Funded since 2023, Available at: https://gepris.dfg.de/gepris/projekt/524609036

[cit125] Wu F., Chen D., Niu Q., Xiao X. (2024). Sustain. Chem. Pharm..

[cit126] Elser J. J., Call D. F., Deaver J. A., Duckworth O. W., Mayer B. K., McLamore E., Rittmann B., Mahmood M., Westerhoff P. (2024). Curr. Opin. Biotechnol..

